# The *Mycobacterium tuberculosis* Rv2745c Plays an Important Role in Responding to Redox Stress

**DOI:** 10.1371/journal.pone.0093604

**Published:** 2014-04-04

**Authors:** Amanda McGillivray, Nadia Abrahams Golden, Uma Shankar Gautam, Smriti Mehra, Deepak Kaushal

**Affiliations:** 1 Divisions of Bacteriology and Parasitology, Tulane National Primate Research Center, Covington, Louisiana, United States of America; 2 Department of Microbiology and Immunology, Tulane University School of Medicine, New Orleans, Louisiana, United States of America; 3 Division of Microbiology, Tulane National Primate Research Center, Covington, Louisiana, United States of America; University of KwaZulu-Natal, South Africa

## Abstract

Tuberculosis (TB), caused by *Mycobacterium tuberculosis* (*Mtb*), is the leading cause of death from an infectious disease worldwide. Over the course of its life cycle *in vivo*, *Mtb* is exposed to a plethora of environmental stress conditions. Temporal regulation of genes involved in sensing and responding to such conditions is therefore crucial for *Mtb* to establish an infection. The Rv2745c (*clgR*) gene encodes a Clp protease gene regulator that is induced in response to a variety of stress conditions and potentially plays a role in *Mtb* pathogenesis. Our isogenic mutant, *Mtb*:ΔRv2745c, is significantly more sensitive to *in vitro* redox stress generated by diamide, relative to wild-type *Mtb* as well as to a complemented strain. Together with the fact that the expression of Rv2745c is strongly induced in response to redox stress, these results strongly implicate a role for ClgR in the management of intraphagosomal redox stress. Additionally, we observed that redox stress led to the dysregulation of the expression of the σ^H^/σ^E^ regulon in the isogenic mutant, *Mtb*:ΔRv2745c. Furthermore, induction of *clgR* in *Mtb* and *Mtb*:ΔRv2745c (comp) did not lead to Clp protease induction, indicating that *clgR* has additional functions that need to be elucidated. Our data, when taken together with that obtained by other groups, indicates that ClgR plays diverse roles in multiple regulatory networks in response to different stress conditions. In addition to redox stress, the expression of Rv2745c correlates with the expression of genes involved in sulfate assimilation as well as in response to hypoxia and reaeration. Clearly, the *Mtb* Rv2745c-encoded ClgR performs different functions during stress response and is important for the pathogenicity of *Mtb in-vivo*, regardless of its induction of the Clp proteolytic pathway.

## Introduction

One third of the population is infected with *Mycobacterium tuberculosis* (*Mtb*), the causative agent of tuberculosis (TB) [1]. TB is the leading cause of death worldwide from an infectious disease and is responsible for approximately 1.4 million deaths annually [1]. Unfortunately, the current vaccine, Bacillus Calmette-Guérine (BCG) vaccine, has a low protective efficacy against adult pulmonary TB [2], [3]. Infected individuals require a long treatment regimen, ranging from 6 to 12 months that involve serious side effects leading to noncompliance, and ultimately the development of resistance. Consequently, understanding the interplay between host and pathogen becomes increasingly important in order to develop alternative treatments and more effective vaccines.

One critical step in *Mtb* pathogenesis is the ability of *Mtb* to reside within the macrophage (MΦ) phagolysosome, as infected alveolar macrophages (AMΦ) are responsible for antigen presentation to CD4^+^ T cells during *Mtb* infection [4], [5]. Residence within the phagolysosome leads to exposure of a plethora of environmental stressors, such as reactive nitrogen species (RNS) and reactive oxygen intermediates (ROI), low pH, and hydrolases, that *Mtb* must be able to sense and respond to in order to establish an infection [6]–[9]. Thus, the temporal regulation of genes involved in sensing and responding to environmental stressors becomes increasingly important for understanding the ability of *Mtb* to take residence within the host MΦ. *Mtb* encodes over 200 regulators of transcription including 13 different sigma factors and is well equipped to respond to rapid changes in its environment.

Upon residence within the host MΦ, *Mtb* intracellular proteins are targets of RNS and ROIs [8]. It is likely that these stress conditions lead to changes in protein conformation, such as misfolding and aggregation. Clearance of misfolded and/or aggregated proteins is necessary for maintenance of protein homeostasis, which is also crucial for infection establishment. One *Mtb* gene that may play a role in this process is Rv2745c (*clgR*), which encodes a Clp protease gene regulator that is induced under a variety of stress conditions [10]–[12]. The expression of Rv2745c appears to be under the control of either σ^E^ or σ^H^, or both. However, the activation of Rv2745c and its role in signaling cascades may be context dependent, implicating additional moonlighting functions for *clgR* in response to different environmental stressors. To study the role of Rv2745c in *Mtb* pathogenesis, we generated an isogenic mutant, *Mtb*:ΔRv2745c, using allelic exchange. Furthermore, we complemented the isogenic mutant to generate a *Mtb*:ΔRv2745c (comp) strain. We analyzed the growth and survival phenotype of these strains, along with wild-type *Mtb*, in response to treatment with a thiol-oxidative agent, diamide, for up to 90 minutes. Further, by subjecting RNA from each strain and untreated controls to comparative transcriptomics, we analyzed the mechanisms by which the Rv2745c gene-product may help *Mtb* respond to redox stress. Our results, presented as part of this investigation, along with those from other groups, indicate that ClgR may fulfill diverse functions in response to the multitude of stress conditions that induce its expression. As such, the roles of *clgR* in signaling cascades in response to stressors is not yet fully defined, thus to attribute clp protease gene regulation as the sole function of Rv2745c is inaccurate.

## Materials and Methods

### Bacterial Strains and Culture Conditions

Liquid cultures of *Mtb* CDC1551 (referred to as *Mtb*), *Mtb*:ΔRv2745c, and *Mtb*:ΔRv2745c (comp) were grown in Middlebrook 7H9 broth (BD Diagnostic Systems) supplemented with 0.1% glycerol, 0.05% Tween-80, 10% Albumin Dextrose Catalase (ADC). Selective antibiotics were used for culturing the isogenic mutant [75 μg/mL hygromycin B (hygro) (Roche Applied Sciences)] as well as the complemented strain [hygro+50 μg/mL kanamycin (kan) (Sigma)].

### Allelic Exchange

Bacteriophage supernatant containing ΔRv2745c::HygR was a kind gift from of Drs William R Jacobs, Jr. and Michelle Larsen, Albert Einstein College of Medicine, Bronx, NY. The supernatant was used to transduce *Mtb* CDC1551 to generate the mutant, *Mtb*:ΔRV2745c following previously published protocols [13].

### Complemented Strain Generation

Rv2745c coding sequence and an additional 481 base pairs upstream were amplified such that they contained *NdeI* and *PacI* restriction sites and cloned into *NdeI* and *PacI* (New England Biolabs) digested pSCW38 integration vector [14], [15]. Following transformation into *E. coli* DH5α, the plasmid was screened for the correct insertion via *NdeI* and *PacI* double digest, PCR screening, and DNA sequencing. After insertion verification, the plasmid was electroporated into *Mtb*:ΔRv2745c to generate *Mtb*:ΔRv2745c (comp) as previously published [13].

### PCR

PCR was performed as per manufacturers’ instructions using the GC-RICH PCR System (Roche Applied Science). PCR was performed using an initial denaturation for 3 min at 95°C followed by 10 cycles of: 30 s at 95°C, 30 s at 65°C, 45 s at 72°C; then 25 cycles of: 95°C, 30 s at 65°C, 45 s (or 1 min depending upon the amplified region) at 72°C; a final elongation for 7 min at 72°C, and then stopped at 4°C. Primer sequences are listed in Table S1 (ST1).

### DNA Extraction

*Mtb* strains were cultured to late exponential phase (OD_260_ 0.8–1.0) in ADC supplemented Middlebrook 7H9 media containing 0.1% Tween-80. Briefly, cells were lysed at 37°C overnight in Lysis Buffer (50 mM Tris, pH 8.0; 150 mM NaCl;10 mM EDTA, pH 8.0; 0.5% SDS) containing Proteinase K (20 μg/mL). Bead beating and subsequent phenol extraction was employed as previously described [14]. DNA concentration was determined using Nanodrop 2000 (ThermoScientific).

### Southern Blot

Southern Blot was performed as previously described by Manganelli, *et al*. and Wang, *et al*. using *NcoI* digested genomic DNA and biotin labeled DNA probes [16], [17].

### Diamide Disc Diffusion Assay

The susceptibility of all three strains were compared via diamide disc diffusion assay as previously published [16].

### In vitro Diamide Treatment

Cultures were grown to mid-log phase (OD_260_ 0.39–0.45) without antibiotics. At time zero, 25 mL of culture was removed for RNA extraction. Upon time zero, cultures were treated with a final concentration of 10 mM diamide as previously described [14]. After treatment, 25 mL of culture was removed for each time point, t = 30, 60, and 90 min. Absorbance readings were taken at each time point.

### Western Blot

Whole cell lysates were extracted from diamide treated cultures at t = 60 minutes post-diamide treatment. A total amount of 10 μg protein was run on an 18% Tris-glycine gel. Protein was transferred and blotted as previously described, with the following changes: anti-Rv2745c antibody was used at a dilution of 1∶500 and goat anti-rabbit was used at a dilution of 1∶200 [14].

### Bacterial RNA Extraction

25 mL of culture was used to extract RNA upon cell lysis via the Trizol bead beater method and phenol extraction [14]. RNA concentrations were quantified using a Nanodrop 2000 (NanoDrop Technologies).

### DNA Microarrays and RT-PCR

*Mtb* specific DNA microarrays (MYcroarrays, Biodiscovery Llc.) were used to compare transcriptome-wide responses in *Mtb*, the mutant and the complemented strains to redox stress by diamide. Detailed protocols for array procedures have been described earlier [10], [14], [16]. Genes were considered to have a perturbed expression level if they exhibited a 2- or a 4-fold higher or lower expression in the mycobacterial strain (wild-type, mutant or the complemented strain) at a given time point, relative to control samples in each of the three biological replicate arrays and in every technical replicate spot on each array. Raw and processed microarray data has been submitted to the Gene Expression Omnibus and can be retrieved using the GEO platform number GPL18320. For real-time (RT) PCR, RNA was treated with DNase as previously described [14]. RNA was reverse transcribed following the manufacturers’ instructions using the High Capacity RNA-to-cDNA Kit (Applied Biosystems) [14]. RT-PCR was performed as per manufacturers’ instructions using Power SYBR Green PCR Master Mix (Applied Biosystems) and as previously described [14]. Expression levels were normalized to sigA levels. For primer sequences, see ST1.

### Statistical Analysis

Statistical analyses were performed using an ANOVA using GraphPad Prism. Microarray statistical analyses were performed using a *t* test script in the Spotfire DecisionSite/S^+^ Array Analyzer.

### Regulatory Compliance

The investigators received approval from the Tulane Institutional Biosafety Committee for all procedures involving *Mtb*.

## Results

### Isogenic Mutant and Complemented Strain Generation

In order to better understand the role played by the product of the Rv2745c gene in the management of host stress during *Mtb* infection, we generated an isogenic mutant in this gene, using *Mtb* CDC1551 as the parental strain. Allelic exchange was employed to generate the isogenic mutant, *Mtb*:ΔRv2745c [13]. Upon selection of isolated colonies from hyg containing plates, genomic DNA isolation and subsequent PCR screening confirms that Rv2745c was successfully replaced with a hygromycin resistant cassette (hyg^r^) upon transduction of *Mtb* with ΔRv2745c::HygR bacteriophage lysate (Figure S1a & b). Replacement of Rv2745c with hyg^r^ was further confirmed via sequencing (data not shown) and Southern Blot (data not shown). Upon generation of the isogenic mutant, an integration vector, pSCW35, containing Rv2745c, was used to complement the deletion mutant, generating *Mtb*:ΔRv2745c (comp). Since the exact location of the promoter element(s) for Rv2745c is unknown, 481 base pairs upstream of Rv2745c on the coding strand were included in the integration vector as this contains both the intergenic region as well as base pairs within the adjacent upstream gene. Upon selection of isolated colonies for genomic DNA isolation, PCR screening identified several candidates that had successful integration of Rv2745c into the *att* site, which was further confirmed via sequencing (Figure S1c and data not shown). To confirm that ClgR protein levels were restored, Rv2745c was induced using diamide treatment and clgR levels were compared between the complemented and wild-type strains via Western Blot (Figure S2). After screening, we selected one out of several of the isolated colonies that had ClgR levels comparable to that of the wild-type *Mtb* upon induction via diamide treatment (Figure S2).

### Diamide Susceptibility

**Figure 1:**
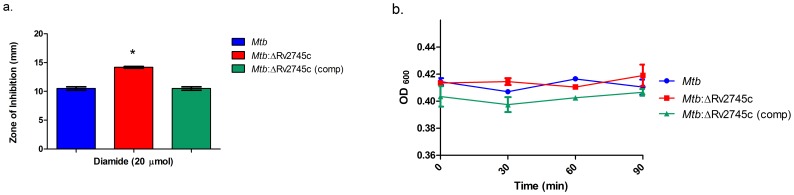
Diamide Susceptibility. a.) Disc diffusion assay was performed using discs containing 20 μmol diamide. The zone of inhibition of *Mtb*:ΔRv2745c (red bar) is significantly larger when compared to both *Mtb* (blue bar) and *Mtb*:ΔRv2745c (comp) (green bar) indicating that the isogenic mutant is more susceptible to redox stress. n = 3. *p<0.001 b.) OD graph of diamide treated cultures. In the initial stages of treatment, there is no significant difference in growth between the different groups.

The current understanding of how Rv2745c contributes to *Mtb* stress responses is largely unknown; specifically, the phenotypic changes associated with loss of Rv2745c function are unknown, as this has not been studied. The expression of Rv2745c is significantly induced in *Mtb* by redox stress *in-vitro.* Hence, upon successful generation of the isogenic mutant and complemented strain, phenotypic changes associated with deletion of Rv275c were assessed via diamide disc diffusion assay. The *Mtb*:ΔRv2745c mutant was more sensitive to redox stress via diamide treatment relative to *Mtb* and *Mtb*:ΔRv2745c (comp), as the zone of inhibition was significantly larger for the isogenic mutant when compared to wild-type and the complemented strains (Figure 1a). Prolonged exposure to redox stress leads to cell death, however when comparing the early response there is not a significant difference between strains (Figure 1b). Thus, it is the initial disruption of signaling cascades that ultimately lead to higher levels of cell death in the isogenic mutant at later time points.

### Transcriptomic Changes Post-Diamide Treatment

**Figure 2:**
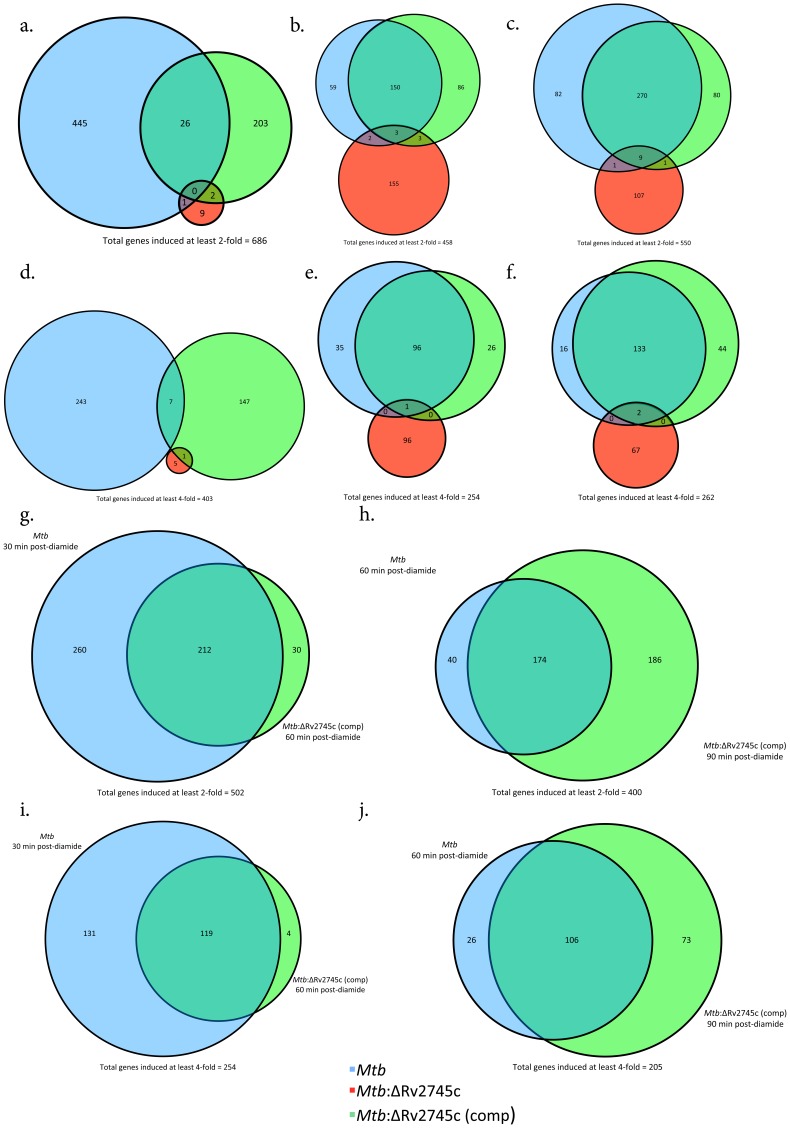
Venn Diagrams of Diamide Induced Genes. Venn diagrams describe the extent of overlap between gene-expression upon diamide treatment in *Mtb* (blue circles), *Mtb*:ΔRv2745c (red circles) and *Mtb*:ΔRv2745c (comp) (green circles). Genes induced at least two-fold at: a.) 30, b.) 60, and c.) 90 min post-diamide treatment are shown. Genes induced at least four-fold at: d.) 30, e.) 60, and f.) 90 min post-diamide treatment. g–j.): Delayed response of *Mtb*:ΔRv2745c (comp). g.) Genes induced at least two-fold comparing *Mtb* at 30 min to *Mtb*:ΔRv2745c (comp) at 60 min and h.) Genes induced at least two-fold comparing *Mtb* at 60 min to *Mtb*:ΔRv2745c (comp) at 90 min. i.) Genes induced at least four-fold comparing *Mtb* at 30 min to *Mtb*:ΔRv2745c (comp) at 60 min. j.) Genes induced at least four-fold comparing *Mtb* at 60 min to *Mtb*:ΔRv2745c (comp) at 90 min. n = 3.

Rv2745c is predicted to encode a transcriptional regulator. Although the expression of the Rv2745c gene is induced by a variety of *in-vitro* stress conditions, the role played by its gene-product in regulating downstream signaling cascades is poorly understood [10], [18]. Thus, Rv2745c expression is induced in *Mtb* upon redox stress by diamide [14]; by membrane damage due to SDS [19] and thioridazine [20], as well as by hypoxia and reaeration [12]. Reaeration and hypoxia but not diamide and thioridazine appear to result in the induction of Clp protease genes *clp*P1, *clp*P2 and *clp*C1. This poses a surprising conundrum and indicates that the product of Rv2745c gene performs different functions in response to host stress, some of which do not require the deployment of the ATP-dependent Clp protease system [10], [11], [18], [21]. We have therefore taken the approach of studying Rv2745c expression and phenotype in a variety of *in-vitro* and *in-vivo* conditions, one at a time, to clearly delineate the role played by Rv2745c-encoded protein. Here we studied expression changes in *Mtb*, the *Mtb*:ΔRv2745c mutant and the complemented strain, in response to diamide stress, as the *Mtb*:ΔRv2745c mutant is clearly susceptible to this condition. We analyzed genome-wide transcriptome responses in *Mtb*, the mutant and the complemented strain to diamide over the course of a 90-minute time period with 30 min intervals. All comparisons were performed relative to untreated controls of the representative strain (Figure 2a).

A significantly higher perturbation in global gene-expression was observed in response to diamide treatment in *Mtb* as well as the complemented strain, rather than the mutant. Hence, none of the 686 genes whose expression was induced by at least two-fold in both *Mtb* and the complemented strain 30 min post-diamide treatment (t = 30), exhibited induction in the mutant (Figure 2a). In contrast, a greater degree of overlap existed between *Mtb* and the complemented strain. Of the 471 and 231 genes whose expression was respectively induced in *Mtb* and the complemented strain, 26 of the genes exhibited overlapping induction. The expression of only one gene overlapped between *Mtb* and the mutant and that of only two overlapped between the complemented strain and the mutant at this early time point (Figure 2a). At t = 30, the expression of not a single gene was induced >two-fold in each of the three strains (Figure 2a).

At the t = 60 min post-diamide treatment, the expression of 458 genes was induced >two-fold in either of the strains, *Mtb* and *Mtb*:ΔRv2745c (comp). The extent of overlap between *Mtb* and the complemented strain increased significantly at this time. Of the 213 and 252 genes whose expression was induced >two-fold in *Mtb* and the complemented strain respectively, 153 (∼72% and 61% respectively) overlapped (Figure 2b). On the other hand, of the 163 genes that exhibited induced expression by least two-fold in the isogenic mutant at t = 60 min post-diamide treatment, only eight overlapped with *Mtb* as well as the complemented strain. At this time point, the expression of only three genes was induced in a shared manner amongst all three strains (Figure 2b). These patterns are similar to that of t = 90 min post-diamide treatment, with 270 genes out of 362 whose expression was induced >two-fold in *Mtb* and 360 whose expression was comparably induced in the complemented strain being induced in an overlapping manner (Figure 2c). Of particular note, a majority of the genes that are induced at least two-fold in the isogenic mutant at both 60 and 90 minutes post-diamide treatment were not shared at each time point.

When we only considered genes induced at least four-fold, the expression of 403 genes was induced at the earliest time point, t = 30 min post-diamide, in either of the strains, wild-type and complemented strain, but none of these genes were shared amongst all three (Figure 2d). At this level of higher stringency, >75% of all genes whose expression was induced at both t = 60 and 90 min post-diamide time points overlapped in *Mtb* as well as the complemented strain. The expression of none of these genes was induced in the *Mtb*:ΔRv2745c mutant (Figure 2e and 2f). We performed quantitative RT-PCR and were able to validate most of the results obtained from the DNA microarray format (not shown).

Taken together these results implicate that the transcriptional response of the *Mtb*:ΔRv2745c mutant to diamide is significantly different from *Mtb* as well as the complemented strain. Hence, Rv2745c plays a role in facilitating the signaling cascades that are required for proper regulation of the redox response. Further, the complemented strain exhibited phenotypic complementation of the response to diamide stress at the t = 60 min and the t = 90 min, but not the t = 30 min time point.

We therefore hypothesized that there may be a delay in the onset of the response of the various regulatory networks in the complemented strain, relative to the wild type strain. We therefore systematically compared the response of *Mtb* at different times to the complemented strain at not the comparable but the subsequent time point. Consistent with our hypothesis, we observed that >87% of all genes with >two-fold expression in the complemented strain at t = 60 min overlapped with those induced in *Mtb* at the previous (t = 30 min) time point; similarly >95% of all genes with >four-fold expression in the complemented strain at t = 60 min overlapped with those induced in *Mtb* at t = 30 min. A high degree of overlap was also present amongst genes whose expression was induced >two- or four-fold in the complemented strain at t = 90 min and *Mtb* at t = 60 min, again suggesting delayed induction of regulatory networks in the former strain (Figure 2g & i and Figure 2h & j).

**Figure 3:**
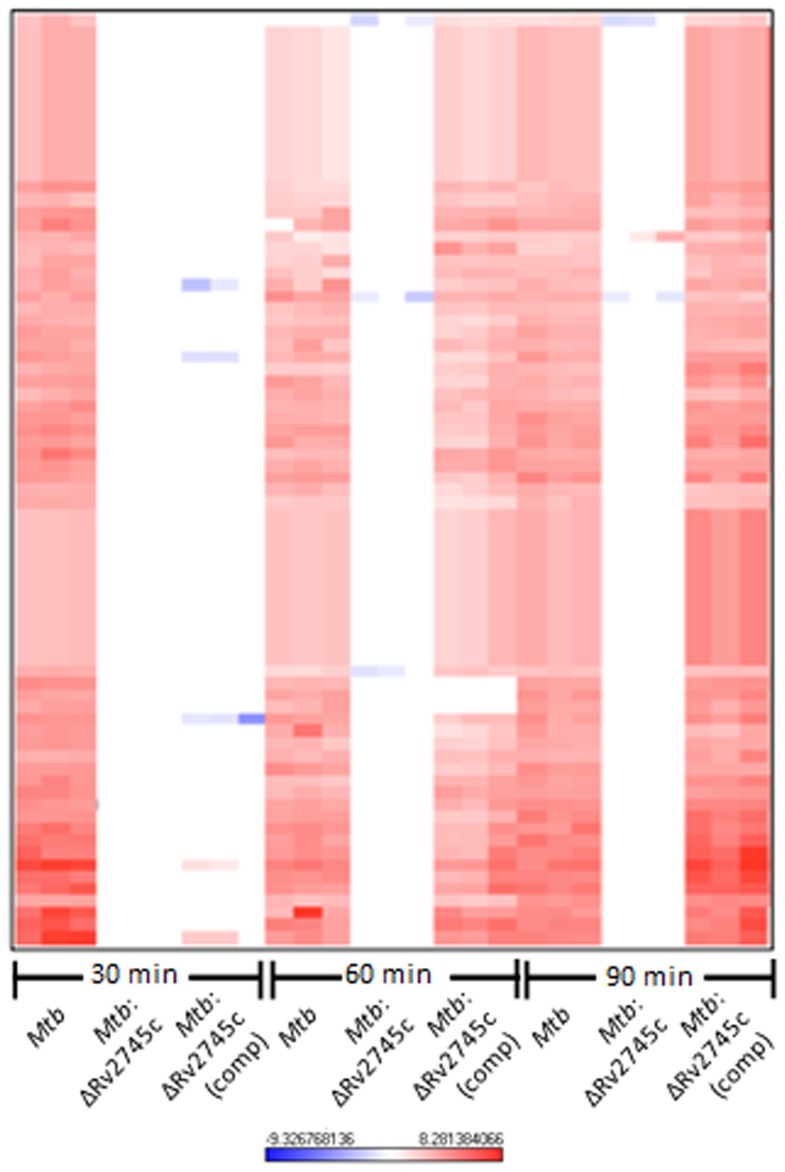
Diamide Induced Genes. The heat map show results of unsupervised hierarchical clustering focusing on the genes with the highest magnitude of change. A majority of genes induced by diamide treatment in *Mtb* are also induced in *Mtb*:ΔRv2745c (comp) by 60 minutes and not induced in *Mtb*:ΔRv2745c. Genes that are induced in *Mtb* are also induced in *Mtb*:ΔRv2745c (comp), whereas there is no change in expression levels in the isogenic mutant indicating that Rv2745c plays a the redox response. n = 3. Red color indicates induction while blue color indicates repression, relative to the control channel. The intensity of each color corresponds to the magnitude.

**Figure 4:**
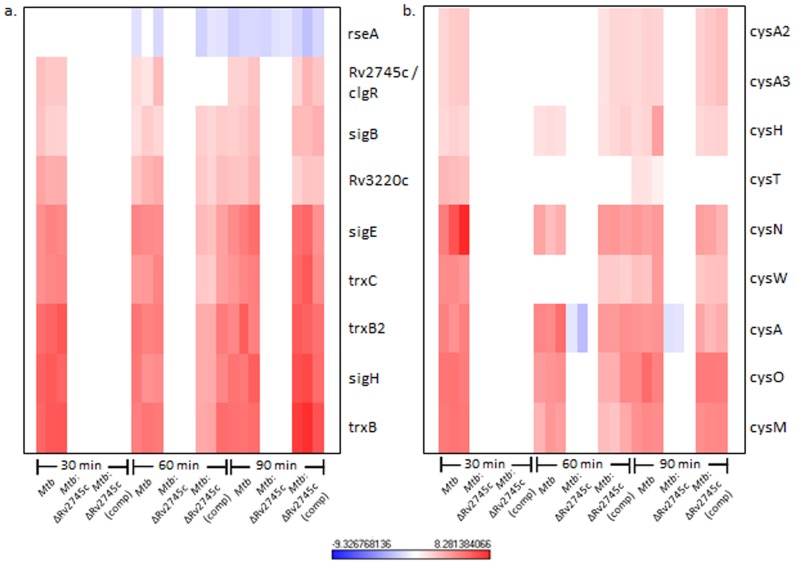
σ^H^ Regulon and Cysteine Pathway Induction. Heat maps show results of unsupervised hierarchical clustering focusing on the genes with the highest magnitude of change. a.) σ^H^ regulon and the b.) cysteine pathway are induced in *Mtb* and *Mtb*:ΔRv2745c (comp), whereas there is no change in expression levels in the isogenic mutant indicating that Rv2745c plays a role in these regulatory networks, via direct or indirect regulation. n = 3. Red color indicates induction while blue color indicates repression, relative to the control channel. The intensity of each color corresponds to the magnitude.

Next, we used hierarchical clustering to identify the specific genes and gene-families with perturbed expression levels in *Mtb* and the complemented strain, relative to the mutant, in response to redox stress. The transcriptional profiles associated with the isogenic mutant were distinct from those observed for both *Mtb* and *Mtb*:ΔRv2745c (comp) (Figure 3, Figure 4a & b). Of the genes induced to the highest levels in the wild type strain, there was no change in expression levels of the isogenic mutant when compared to the untreated control (Figure 3). Again, the delayed response of the complemented strain was reflected in the heat maps, showing that the expression pattern seen in the wildtype strain is restored by 60 min post-diamide treatment in the complemented strain (Figure 3, Figure 4a & b).

The regulatory networks that were generally induced in *Mtb* and the complemented strain, following redox stress, expectedly belonged to the σ^H^-regulon [14]. Surprisingly however, the expression of a large majority of these genes was disrupted in the *Mtb*:ΔRv2745c mutant (Figure 3, Figure 4a). In addition to positively reinforcing the induction of the σ^H^ regulon, these genes play a role in the stress response and detoxification and cysteine biosynthesis (Figure 4a & b) [10], [22]. The cysteine biosynthetic pathway comprises of two arms critical for response to redox stress [23], [24]. The alternative cysteine pathways utilizes thiocarboxylates for cysteine synthesis, which are more resistant to oxidative stress – the type of environment found within the MΦ [23]. Genes encoding CysM and CysO, which are part of the alternative cysteine pathway, exhibit up regulation upon oxidative stress in both *Mtb* and *Mtb*:ΔRv2745c (comp). However this expression pattern is disrupted in *Mtb*:ΔRv2745c (Figure 4b) [23], [25].

While the up regulation of genes within the σ^H^ regulon in *Mtb* and *Mtb*:ΔRv2745c (comp) is expected, as Rv2745c is activated downstream of σ^H^ in response to diamide (Figure 4a) [10], [22], the complete disruption of this regulatory pathway in the mutant is surprising and suggests that the Rv2745c-encoded protein may perform alternative functions in addition to activating ATP-dependent Clp proteases, the expression of which does not occur upon diamide treatment [16].

Additionally, transcription levels of several heat shock protein coding genes were also disrupted in *Mtb*:ΔRv2745c. Thus, *htp*X and *htr*A expression was up regulated in *Mtb* at all time points, but remain at basal levels in *Mtb*:ΔRv2745c (Tables 1, 2, 3, 4, 5, 6, 7, 8, 9). The induction of these heat shock genes in both the wild-type and the complemented strains is again not surprising, since their expression is known to be induced in a σ^H^ dependent manner [14]. The role of clgR in activation of heat shock proteins is further supported by Mehra, *et al*., who found that induction of Rv2745c under a tetracycline promoter lead to higher expression levels of heat shock proteins when compared to un-induced samples [10].

**Table 1:** *Mtb* 30 min post-diamide treatment.

Function	MT #	Symbol	Description	Rv#	M1	M2	M3	AverageM Value	ExpressionFold Change
Heat Shock	MT0397	clpB	ATP-dependent Clp protease, ATP-binding subunit	Rv0384c	3.135	3.814	3.920	3.623	12.320
	MT0265	hsp	heat shock protein, HSP20 family	Rv0251c	4.291	4.419	4.201	4.304	19.749
	MT0589	htpX	heat shock protein HtpX	Rv0563	1.558	1.859	1.402	1.606	3.045
	MT0365	dnaK	dnaK protein	Rv0350	2.964	3.292	3.109	3.122	8.703
	MT0367	dnaJ1	dnaJ protein	Rv0352	2.890	2.984	2.923	2.932	7.634
Transcription	MT2816	clgR	DNA-binding protein, putative	Rv2745c	2.137	2.537	2.170	2.281	4.862
	MT2783	sigB	RNA polymerase principal sigma factor SigB	Rv2710	1.825	2.413	1.768	2.002	4.006
	MT1259	sigE	RNA polymerase sigma-70 factor, ECF subfamily	Rv1221	4.864	4.327	4.614	4.602	24.282
	MT3320	sigH	RNA polymerase sigma-70 factor, ECF subfamily	Rv3223c	6.455	6.134	6.033	6.208	73.904
	MT4030	sigM	RNA polymerase sigma-70 factor, ECF subfamily	Rv3911	1.277	2.191	1.760	1.742	3.346
	MT1960	furA	ferric uptake regulation protein	Rv1909c	2.369	3.518	3.244	3.044	8.247
	MT1260	rseA	hypothetical protein	Rv1222	4.990	5.047	4.952	4.996	31.918
	MT3316		sensor histidine kinase	Rv3220c	3.158	3.583	3.231	3.324	10.014
Transport	MT2468	cysA1	sulfate ABC transporter, ATP-binding protein	Rv2397c	4.467	4.511	3.816	4.265	19.220
	MT2470	cysT	sulfate ABC transporter, permease protein	Rv2399c	2.572	2.203	2.352	2.375	5.189
	MT2469	cysW	sulfate ABC transporter, permease protein	Rv2398c	4.076	3.767	4.115	3.986	15.846
	MT1519		ABC transporter, ATP-binding protein	Rv1473	3.819	3.999	3.820	3.879	14.716
	MT2471	subI	sulfate ABC transporter, sulfate-binding	Rv2400c	2.003	2.246	2.398	2.216	4.645
Detoxification	MT2719	cadI	conserved hypothetical protein	Rv2641	6.218	7.605	6.767	6.863	116.434
	MT1517	trxB1	thioredoxin	Rv1471	6.548	5.952	6.621	6.374	82.915
	MT4032	trxB2	thioredoxin reductase	Rv3913	6.052	5.673	6.619	6.114	69.286
	MT4033	trxC	thioredoxin	Rv3914	4.676	4.360	4.830	4.622	24.620
	MT1959	katG	catalase-peroxidase	Rv1908c	1.313	1.553	1.786	1.551	2.929
Molybdopterin biosynthesis	MT2528	mobA	molybdopterin-guanine dinucleotide biosynthesis	Rv2453c	2.806	3.000	2.753	2.853	7.226
	MT3301	moeB1	HesA/MoeB/ThiF family protein	Rv3206c	4.683	4.840	4.753	4.759	27.069
Sulfate metabolism	MT1377	cysM	cysteine synthase	Rv1336	4.728	4.767	4.930	4.808	28.013
	MT1324	cysN	sulfate adenylate transferase, subunit 1	Rv1286	4.620	7.578	6.087	6.095	68.357
	MT1323	cysD	sulfate adenylate transferase, subunit 2	Rv1285	3.022	3.410	3.629	3.354	10.224
	MT1376.1	cysO	conserved hypothetical protein	Rv1335	4.767	4.635	4.658	4.687	25.825
	MT0837	cysA2	thiosulfate sulfurtransferase	Rv0815c	1.511	1.891	1.856	1.753	3.370
	MT3199	cysA3	thiosulfate sulfurtransferase	Rv3117	1.743	1.976	1.829	1.850	3.604
	MT2462	cysH	phosphoadenosine phosphosulfate reductase	Rv2392	1.365	1.453	1.627	1.481	2.792
Cell wall associated	MT0856	lpqQ	hypothetical protein	Rv0835	1.244	1.123	1.351	1.239	2.361
	MT0870	lpqS	hypothetical protein	Rv0847	2.824	2.865	3.236	2.975	7.861
	MT1379	murI	glutamate racemase	Rv1338	4.430	3.888	4.418	4.245	18.967

Genes induced and repressed by functional category at 30 minutes post-diamide treatment in wild-type. n = 3. MT # and Rv # denote the CDC1551 and the H37Rv gene IDs, respectively.

**Table 2:** *Mtb*:ΔRv2745c 30 min post-diamide treatment.

Function	MT #	Symbol	Description	Rv#	M1	M2	M3	AverageM Value	ExpressionFold Change
Heat Shock	MT0397	clpB	ATP-dependent Clp protease, ATP-binding subunit	Rv0384c	ND	ND	ND	–	–
	MT0265	hsp	heat shock protein, HSP20 family	Rv0251c	ND	ND	ND	–	–
	MT0589	htpX	heat shock protein HtpX	Rv0563	ND	ND	ND	–	–
	MT0365	dnaK	dnaK protein	Rv0350	ND	ND	ND	–	–
	MT0367	dnaJ1	dnaJ protein	Rv0352	ND	ND	ND	–	–
Transcription	MT2816	clgR	DNA-binding protein, putative	Rv2745c	ND	ND	ND	–	–
	MT2783	sigB	RNA polymerase principal sigma factor SigB	Rv2710	ND	ND	ND	–	–
	MT1259	sigE	RNA polymerase sigma-70 factor, ECF subfamily	Rv1221	ND	ND	ND	–	–
	MT3320	sigH	RNA polymerase sigma-70 factor, ECF subfamily	Rv3223c	ND	ND	ND	–	–
	MT4030	sigM	RNA polymerase sigma-70 factor, ECF subfamily	Rv3911	ND	ND	ND	–	–
	MT1960	furA	ferric uptake regulation protein	Rv1909c	ND	ND	ND	–	–
	MT1260	rseA	hypothetical protein	Rv1222	ND	ND	ND	–	–
	MT3316		sensor histidine kinase	Rv3220c	ND	ND	ND	–	–
Transport	MT2468	cysA1	sulfate ABC transporter, ATP-binding protein	Rv2397c	ND	ND	ND	–	–
	MT2470	cysT	sulfate ABC transporter, permease protein	Rv2399c	ND	ND	ND	–	–
	MT2469	cysW	sulfate ABC transporter, permease protein	Rv2398c	ND	ND	ND	–	–
	MT1519		ABC transporter, ATP-binding protein	Rv1473	ND	ND	ND	–	–
	MT2471	subI	sulfate ABC transporter, sulfate-binding	Rv2400c	ND	ND	ND	–	–
Detoxification	MT2719	cadI	conserved hypothetical protein	Rv2641	ND	ND	ND	–	–
	MT1517	trxB1	thioredoxin	Rv1471	ND	ND	ND	–	–
	MT4032	trxB2	thioredoxin reductase	Rv3913	ND	ND	ND	–	–
	MT4033	trxC	thioredoxin	Rv3914	ND	ND	ND	–	–
	MT1959	katG	catalase-peroxidase	Rv1908c	ND	ND	ND	–	–
Molybdopterin biosynthesis	MT2528	mobA	molybdopterin-guanine dinucleotide biosynthesis	Rv2453c	ND	ND	ND	–	–
	MT3301	moeB1	HesA/MoeB/ThiF family protein	Rv3206c	ND	ND	ND	–	–
Sulfate metabolism	MT1377	cysM	cysteine synthase	Rv1336	ND	ND	ND	–	–
	MT1324	cysN	sulfate adenylate transferase, subunit 1	Rv1286	ND	ND	ND	–	–
	MT1323	cysD	sulfate adenylate transferase, subunit 2	Rv1285	ND	ND	ND	–	–
	MT1376.1	cysO	conserved hypothetical protein	Rv1335	ND	ND	ND	–	–
	MT0837	cysA2	thiosulfate sulfurtransferase	Rv0815c	ND	ND	ND	–	–
	MT3199	cysA3	thiosulfate sulfurtransferase	Rv3117	ND	ND	ND	–	–
	MT2462	cysH	phosphoadenosine phosphosulfate reductase	Rv2392	ND	ND	ND	–	–
Cell wall associated	MT0856	lpqQ	hypothetical protein	Rv0835	ND	ND	ND	–	–
	MT0870	lpqS	hypothetical protein	Rv0847	ND	ND	ND	–	–
	MT1379	murI	glutamate racemase	Rv1338	ND	ND	ND	–	–

Genes induced and repressed by functional category at 30 minutes post-diamide treatment in the isogenic mutant. n = 3. MT # and Rv # denote the CDC1551 and the H37Rv gene IDs, respectively.

**Table 3:** *Mtb*:ΔRv2745c (comp) 30 min post-diamide treatment.

Function	MT #	Symbol	Description	Rv#	M1	M2	M3	AverageM Value	ExpressionFold Change
Transcription	MT2784	ideR	Transcriptional Regulatory Protein (Repressor and activator)	Rv2711	–	−1.580	−1.508	−1.544	−2.916
	MT1685		purine cyclase-related protein	Rv1647	4.080	2.325	–	3.202	9.206
Transport	MT1907	modC	molybdate uptake ABC-transporter	Rv1859	−2.183	−1.196	−	−1.690	−3.225
Detoxification	MT2967	fdhD	fdhD protein	Rv2899c	−	3.234	2.899	3.066	8.377
Intermediary metabolism	MT3719	ephA	probable epoxide hydrolase	Rv3617	−	−7.782	−1.798	−4.790	−27.670
Cell wall associated	MT1593	lprI	lipoprotein	Rv1541c	−1.287	−1.063	−	−1.175	−2.258

Genes induced and repressed by functional category at 30 minutes post-diamide treatment in the complemented strain. n = 3. MT # and Rv # denote the CDC1551 and the H37Rv gene IDs, respectively.

**Table 4:** *Mtb* 60 min post-diamide treatment.

Function	MT #	Symbol	Description	Rv#	M1	M2	M3	Average M Value	Expression Fold Change
Heat Shock	MT0397	clpB	ATP-dependent Clp protease, ATP-binding subunit	Rv0384c	1.302	1.850	2.051	1.734	3.328
	MT3527	groES	chaperonin, 10 kDa	Rv3418c	−2.060	−2.279	−2.545	−2.295	−4.906
	MT0265	hsp	heat shock protein, HSP20 family	Rv0251c	3.300	3.000	3.550	3.283	9.736
	MT0589	htpX	heat shock protein HtpX	Rv0563	1.085	2.251	2.747	2.028	4.077
	MT0365	dnaK	dnaK protein	Rv0350	2.921	2.712	2.878	2.837	7.145
	MT0367	dnaJ1	dnaJ protein	Rv0352	2.362	2.776	2.447	2.528	5.769
Transcription	MT2816	clgR	DNA−binding protein, putative	Rv2745c	1.044	1.444	2.712	1.734	3.326
	MT2783	sigB	RNA polymerase principal sigma factor SigB	Rv2710	2.010	1.250	1.580	1.613	3.060
	MT1259	sigE	RNA polymerase sigma−70 factor, ECF subfamily	Rv1221	4.580	4.760	4.450	4.597	24.195
	MT3320	sigH	RNA polymerase sigma−70 factor, ECF subfamily	Rv3223c	4.276	5.269	4.498	4.681	25.652
	MT1960	furA	ferric uptake regulation protein		1.730	3.678	2.191	2.533	5.788
	MT1009	mprA	DNA−binding response regulator	Rv0981	−1.566	−2.026	−1.646	−1.746	−3.355
	MT1260	rseA	hypothetical protein	Rv1222	4.570	4.580	4.340	4.497	22.575
	MT3316		sensor histidine kinase	Rv3220c	2.953	2.307	3.289	2.850	7.208
Transport	MT2468	cysA1	sulfate ABC transporter, ATP−binding protein	Rv2397c	4.264	4.103	5.166	4.511	22.801
	MT1519		ABC transporter, ATP−binding protein	Rv1473	3.740	3.333	3.780	3.618	12.275
Detoxification	MT2719	cadI	conserved hypothetical protein	Rv2641	4.500	4.640	5.040	4.727	26.477
	MT1517	trxB1	thioredoxin	Rv1471	5.440	5.030	5.230	5.233	37.618
	MT4032	trxB2	thioredoxin reductase	Rv3913	4.930	5.430	5.120	5.160	35.753
	MT4033	trxC	thioredoxin	Rv3914	3.974	3.897	4.842	4.238	18.865
	MT1959	katG	catalase−peroxidase	Rv1908c	1.840	1.220	1.610	1.557	2.942
	MT3174	fadD13	substrate–CoA ligase	Rv3089	1.805	1.641	1.262	1.569	2.968
Molybdopterin biosynthesis	MT2528	mobA	molybdopterin−guanine dinucleotide biosynthesis	Rv2453c	2.190	1.800	2.740	2.243	4.735
	MT3301	moeB1	HesA/MoeB/ThiF family protein	Rv3206c	4.275	4.684	5.231	4.730	26.538
Sulfate metabolism	MT1377	cysM	cysteine synthase	Rv1336	2.700	3.640	3.410	3.250	9.514
	MT1324	cysN	sulfate adenylate transferase, subunit 1	Rv1286	3.236	2.299	2.838	2.791	6.921
	MT1376.1	cysO	conserved hypothetical protein	Rv1335	3.560	3.760	3.960	3.760	13.548
	MT3199	cysA3	thiosulfate sulfurtransferase	Rv3117	1.190	1.870	3.260	2.107	4.307
	MT2462	cysH	phosphoadenosine phosphosulfate reductase	Rv2392	1.078	1.219	1.122	1.140	2.204
Intermediary metabolism	MT3949	bfrB	ferritin family protein	Rv3841	−1.295	−1.422	−1.739	−1.486	−2.800
Cell wall associated	MT0870	lpqS	hypothetical protein	Rv0847	1.477	1.871	2.347	1.898	3.728
	MT1379	murI	glutamate racemase	Rv1338	2.810	4.610	3.190	3.537	11.605

Genes induced and repressed by functional category at 60 minutes post-diamide treatment in wild-type. n = 3. MT # and Rv # denote the CDC1551 and the H37Rv gene IDs, respectively.

**Table 5:** *Mtb*:ΔRv2745c 60 min post-diamide treatment.

Function	MT #	Symbol	Description	Rv#	M1	M2	M3	Average M Value	Expression Fold Change
Transport	MT2468	cysA1	sulfate ABC transporter, ATP-binding protein	Rv2397c	−2.721	−	−1.053	−1.887	−3.699
	MT1907	modC	ABC transporter, ATP-binding protein	Rv1859	−1.616	−	−3.854	−2.735	−6.657
	MT0951	mntH	transport protein, NRAMP family	Rv0924c	6.500	3.995	1.122	3.872	14.645

Genes induced and repressed by functional category at 60 minutes post-diamide treatment in the isogenic mutant. n = 3. MT # and Rv # denote the CDC1551 and the H37Rv gene IDs, respectively.

**Table 6:** *Mtb*:ΔRv2745c (comp) 60 min post-diamide treatment.

Function	MT #	Symbol	Description	Rv#	M1	M2	M3	Average M Value	Expression Fold Change
Heat Shock	MT0397	clpB	ATP-dependent Clp protease, ATP-binding subunit	Rv0384c	2.454	1.961	2.321	2.245	4.741
	MT3526	groEL1		Rv3417c	1.697	1.664	1.765	1.709	3.269
	MT0265	hsp	heat shock protein, HSP20 family	Rv0251c	2.973	2.698	3.026	2.899	7.459
	MT0589	htpX	heat shock protein HtpX	Rv0563	1.759	1.651	1.804	1.738	3.336
	MT0365	dnaK	dnaK protein	Rv0350	2.158	2.336	3.394	2.629	6.187
	MT0367	dnaJ1	dnaJ protein	Rv0352	2.377	2.301	3.632	2.770	6.821
Transcription	MT2783	sigB	RNA polymerase principal sigma factor SigB	Rv2710	1.894	1.589	2.014	1.832	3.560
	MT1259	sigE	RNA polymerase sigma-70 factor, ECF subfamily	Rv1221	2.737	2.447	3.679	2.954	7.750
	MT3320	sigH	RNA polymerase sigma-70 factor, ECF subfamily	Rv3223c	3.223	3.345	4.739	3.769	13.635
	MT1960	furA		Rv1909c	2.465	2.646	2.811	2.641	6.236
	MT1009	mprA		Rv0981	−1.426	−1.742	−1.638	−1.602	−3.035
	MT1260	rseA	hypothetical protein	Rv1222	2.657	2.646	3.716	3.006	8.034
	MT3316		sensor histidine kinase	Rv3220c	2.100	1.708	2.563	2.124	4.358
Transport	MT2468	cysA1		Rv2397c	3.588	3.563	3.985	3.712	13.107
	MT2469	cysW	sulfate ABC transporter, permease protein	Rv2398c	1.845	1.869	1.635	1.783	3.442
	MT1519			Rv1473	3.022	2.958	3.803	3.261	9.588
	MT2471	subI	sulfate ABC transporter, sulfate−binding	Rv2400c	1.098	1.467	1.011	1.192	2.285
Detoxification	MT2719	cadI		Rv2641	4.175	4.322	6.555	5.017	32.389
	MT1517	trxB1	thioredoxin	Rv1471	3.349	3.605	5.726	4.227	18.725
	MT4032	trxB2	thioredoxin reductase	Rv3913	3.218	3.290	5.195	3.901	14.936
	MT4033	trxC	thioredoxin	Rv3914	2.160	2.018	3.730	2.636	6.216
	MT1959	katG	catalase−peroxidase	Rv1908c	1.713	2.331	2.757	2.267	4.814
Lipid metabolism	MT0882	fadA		Rv0859	−2.372	−1.416	−1.240	−1.676	−3.195
Molybdopterin biosynthesis	MT2528	mobA	molybdopterin−guanine dinucleotide biosynthesis	Rv2453c	2.375	2.000	2.914	2.430	5.388
	MT3301	moeB1	HesA/MoeB/ThiF family protein	Rv3206c	2.481	2.625	3.363	2.823	7.075
Sulfate metabolism	MT1377	cysM	cysteine synthase	Rv1336	2.346	2.104	2.969	2.473	5.552
	MT1324	cysN	sulfate adenylate transferase, subunit 1	Rv1286	3.611	3.698	3.389	3.566	11.841
	MT1376.1	cysO	conserved hypothetical protein	Rv1335	2.797	2.808	4.142	3.249	9.507
	MT0837	cysA2	thiosulfate sulfurtransferase	Rv0815c	1.008	1.467	1.478	1.318	2.493
	MT3199	cysA3	thiosulfate sulfurtransferase	Rv3117	1.516	1.358	1.686	1.520	2.868
	MT2462	cysH	phosphoadenosine phosphosulfate reductase	Rv2392	1.108	1.405	1.696	1.403	2.645
Intermediary metabolism	MT3949	bfrB		Rv3841	−1.440	−1.332	−1.177	−1.316	−2.490
	MT3969	ethA		Rv3854c	−	−1.173	−1.226	−1.200	−2.297
Cell wall associated	MT0870	lpqS	hypothetical protein	Rv0847	1.258	1.157	1.404	1.273	2.416
	MT1379	murI	glutamate racemase	Rv1338	3.322	2.855	3.729	3.302	9.863

Genes induced and repressed by functional category at 60 minutes post-diamide treatment in the complemented strain. n = 3. MT # and Rv # denote the CDC1551 and the H37Rv gene IDs, respectively.

**Table 7:** *Mtb* 90 min post-diamide treatment.

Function	MT #	Symbol	Description	Rv#	M1	M2	M3	Average M Value	Expression Fold Change
Heat Shock	MT0397	clpB	ATP-dependent Clp protease, ATP-binding subunit	Rv0384c	3.003	3.121	3.426	3.183	9.083
	MT0265	hsp	heat shock protein, HSP20 family	Rv0251c	5.176	4.723	5.038	4.979	31.534
	MT0589	htpX	heat shock protein HtpX	Rv0563	1.806	2.009	2.109	1.975	3.930
	MT0365	dnaK	dnaK protein	Rv0350	3.616	3.287	3.971	3.625	12.336
	MT0367	dnaJ1	dnaJ protein	Rv0352	2.993	2.707	3.254	2.985	7.916
Transcription	MT2816	clgR	DNA-binding protein, putative	Rv2745c	1.737	1.731	2.349	1.939	3.835
	MT2783	sigB	RNA polymerase principal sigma factor SigB	Rv2710	2.162	1.906	2.612	2.227	4.680
	MT1259	sigE	RNA polymerase sigma-70 factor, ECF subfamily	Rv1221	5.082	4.539	5.660	5.094	34.147
	MT3320	sigH	RNA polymerase sigma-70 factor, ECF subfamily	Rv3223c	5.271	5.486	6.261	5.673	51.011
	MT4030	sigM	RNA polymerase sigma-70 factor, ECF subfamily	Rv3911	1.286	0.768	1.015	1.023	2.032
	MT1960	furA	ferric uptake regulation protein	Rv1909c	2.279	2.663	2.635	2.525	5.757
	MT2784	ideR	iron-dependent repressor IdeR	Rv2711	1.185	1.088	0.911	1.061	2.087
	MT1009	mprA	DNA-binding response regulator	Rv0981	−1.807	−1.601	−1.578	−1.662	−3.164
	MT1260	rseA	hypothetical protein	Rv1222	5.400	5.001	5.968	5.456	43.910
	MT3316		sensor histidine kinase	Rv3220c	2.304	2.344	2.997	2.548	5.850
Transport	MT2468	cysA1	sulfate ABC transporter, ATP-binding protein	Rv2397c	3.785	3.824	3.643	3.751	13.461
	MT2469	cysW	sulfate ABC transporter, permease protein	Rv2398c	2.201	3.545	2.075	2.607	6.092
	MT1519		ABC transporter, ATP-binding protein	Rv1473	3.679	3.609	3.936	3.741	13.371
	MT2471	subI	sulfate ABC transporter, sulfate-binding	Rv2400c	1.399	1.475	1.462	1.446	2.724
Detoxification	MT2719	cadI	conserved hypothetical protein	Rv2641	6.100	5.606	6.200	5.969	62.619
	MT1517	trxB1	thioredoxin	Rv1471	5.489	5.535	5.724	5.583	47.931
	MT4032	trxB2	thioredoxin reductase	Rv3913	6.348	4.685	4.867	5.300	39.399
	MT4033	trxC	thioredoxin	Rv3914	4.689	4.151	5.029	4.623	24.643
	MT1959	katG	catalase-peroxidase	Rv1908c	1.760	2.116	2.419	2.098	4.282
	MT0179	mce1B	virulence factor mce family protein	Rv0170	−1.355	−1.366	−1.269	−1.330	−2.514
	MT3960	sodA	superoxide dismutase	Rv3846	−	−1.528	−1.841	−1.684	−3.214
Lipid metabolism	MT2303	fabD	malonyl CoA-acyl carrier protein transacylase	Rv2243	1.512	1.672	1.971	1.718	3.290
Molybdopterin biosynthesis	MT2528	mobA	molybdopterin-guanine dinucleotide biosynthesis	Rv2453c	2.598	2.386	2.693	2.559	5.893
	MT3301	moeB1	HesA/MoeB/ThiF family protein	Rv3206c	4.691	4.579	5.222	4.831	28.457
Sulfate metabolism	MT1377	cysM	cysteine synthase	Rv1336	3.860	4.007	4.132	4.000	15.996
	MT1324	cysN	sulfate adenylate transferase, subunit 1	Rv1286	3.646	3.630	3.324	3.533	11.579
	MT1323	cysD	sulfate adenylate transferase, subunit 2	Rv1285	3.377	2.964	3.064	3.135	8.784
	MT1376.1	cysO	conserved hypothetical protein	Rv1335	4.195	4.459	5.209	4.621	24.606
	MT0837	cysA2	thiosulfate sulfurtransferase	Rv0815c	1.307	1.543	1.616	1.489	2.806
	MT3199	cysA3	thiosulfate sulfurtransferase	Rv3117	1.338	1.350	1.649	1.446	2.724
	MT2462	cysH	phosphoadenosine phosphosulfate reductase	Rv2392	1.275	3.358	1.492	2.042	4.117
Cell wall associated	MT0870	lpqS	hypothetical protein	Rv0847	2.474	2.392	2.852	2.573	5.950
	MT1379	murI	glutamate racemase	Rv1338	3.520	3.437	3.730	3.562	11.814

Genes induced and repressed by functional category at 90 minutes post-diamide treatment in wild-type. n = 3. MT # and Rv # denote the CDC1551 and the H37Rv gene IDs, respectively.

**Table 8:** *Mtb*:ΔRv2745c 90 min post-diamide treatment.

Function	MT #	Symbol	Description	Rv#	M1	M2	M3	Average M Value	Expression Fold Change
Transcription	MT4030	sigM	RNA polymerase sigma-70 factor, ECF subfamily	Rv3911	−1.090	−	−1.495	−1.292	−2.449
Transport	MT2468	cysA1	sulfate ABC transporter, ATP-binding protein	Rv2397c	−1.043	−1.266	−	−1.154	−2.226

Genes induced and repressed by functional category 90 minutes post-diamide treatment in the isogenic mutant. n = 3. MT # and Rv # denote the CDC1551 and the H37Rv gene IDs, respectively.

**Table 9:** *Mtb*:ΔRv2745c (comp) 90 min post-diamide treatment.

Function	MT #	Symbol	Description	Rv#	M1	M2	M3	Average M Value	Expression Fold Change
Heat Shock	MT0397	clpB	ATP-dependent Clp protease, ATP-binding subunit	Rv0384c	3.987	4.158	3.667	3.937	15.320
	MT3526	groEL1	chaperonin, 60 kDa	Rv3417c	1.868	1.944	2.967	2.260	4.789
	MT3527	groES	chaperonin, 10 kDa	Rv3418c	1.298	2.344	3.283	2.308	4.952
	MT0265	hsp	heat shock protein, HSP20 family	Rv0251c	5.430	5.139	5.495	5.355	40.917
	MT0589	htpX	heat shock protein HtpX	Rv0563	2.416	2.120	2.321	2.286	4.876
	MT0365	dnaK	dnaK protein	Rv0350	5.646	5.429	4.661	5.245	37.927
	MT0367	dnaJ1	dnaJ protein	Rv0352	4.054	4.365	4.101	4.173	18.042
Transcription	MT2816	clgR	DNA-binding protein, putative	Rv2745c	2.978	1.678	2.261	2.306	4.944
	MT2783	sigB	RNA polymerase principal sigma factor SigB	Rv2710	2.667	2.720	3.112	2.833	7.125
	MT1259	sigE	RNA polymerase sigma-70 factor, ECF subfamily	Rv1221	5.964	5.573	4.292	5.276	38.753
	MT3320	sigH	RNA polymerase sigma-70 factor, ECF subfamily	Rv3223c	7.174	6.878	5.741	6.597	96.836
	MT4030	sigM	RNA polymerase sigma-70 factor, ECF subfamily	Rv3911	1.592	1.395	1.143	1.377	2.597
	MT0017	pknB	serine/threonine protein kinase	Rv0014c	−1.240	−2.333	−1.012	−1.528	−2.884
	MT1960	furA	ferric uptake regulation protein	Rv1909c	3.102	2.845	3.261	3.069	8.393
	MT2784	ideR	iron-dependent repressor IdeR	Rv2711	1.199	1.113	1.074	1.129	2.187
	MT1009	mprA	DNA-binding response regulator	Rv0981	−2.224	−1.402	−1.606	−1.744	−3.349
	MT1260	rseA	hypothetical protein	Rv1222	6.595	6.524	5.785	6.301	78.872
	MT3316		sensor histidine kinase	Rv3220c	2.331	1.731	2.241	2.101	4.290
Transport	MT2468	cysA1	sulfate ABC transporter, ATP-binding protein	Rv2397c	2.478	2.905	3.187	2.857	7.243
	MT2469	cysW	sulfate ABC transporter, permease protein	Rv2398c	2.201	2.229	1.920	2.117	4.337
	MT1519		ABC transporter, ATP-binding protein	Rv1473	5.628	4.510	3.948	4.695	25.911
	MT2471	subI	sulfate ABC transporter, sulfate-binding	Rv2400c	1.735	1.457	1.438	1.543	2.915
Detoxification	MT2719	cadI	conserved hypothetical protein	Rv2641	7.428	6.957	6.975	7.120	139.088
	MT1517	trxB1	thioredoxin	Rv1471	8.175	7.631	6.737	7.514	182.799
	MT4032	trxB2	thioredoxin reductase	Rv3913	6.124	6.487	5.555	6.055	66.508
	MT4033	trxC	thioredoxin	Rv3914	6.542	5.853	4.581	5.659	50.522
	MT1959	katG	catalase-peroxidase	Rv1908c	3.273	3.200	3.162	3.212	9.265
Lipid metabolism	MT1001	accA2	acetyl/propionyl-CoA carboxylase, alpha subunit	Rv0973c	−1.270	−1.101	−1.222	−1.197	−2.293
	MT3350	alkB	alkane-1 monooxygenase	Rv3252c	−1.885	−1.538	−1.399	−1.607	−3.047
	MT0882	fadA	thiolase	Rv0859	−1.413	−1.627	−1.280	−1.440	−2.712
Molybdopterin biosynthesis	MT2528	mobA	molybdopterin-guanine dinucleotide biosynthesis	Rv2453c	3.241	3.688	3.189	3.373	10.360
	MT3301	moeB1	HesA/MoeB/ThiF family protein	Rv3206c	4.233	4.164	4.196	4.198	18.349
Sulfate metabolism	MT1377	cysM	cysteine synthase	Rv1336	4.163	4.227	3.935	4.108	17.246
	MT1324	cysN	sulfate adenylate transferase, subunit 1	Rv1286	3.263	2.650	3.421	3.111	8.641
	MT1323	cysD	sulfate adenylate transferase, subunit 2	Rv1285	2.797	2.243	3.263	2.768	6.811
	MT1376.1	cysO	conserved hypothetical protein	Rv1335	4.680	4.595	4.668	4.648	25.068
	MT0837	cysA2	thiosulfate sulfurtransferase	Rv0815c	1.922	2.300	1.637	1.953	3.872
	MT3199	cysA3	thiosulfate sulfurtransferase	Rv3117	2.059	2.400	1.490	1.983	3.953
	MT2462	cysH	phosphoadenosine phosphosulfate reductase	Rv2392	1.536	1.563	1.349	1.483	2.794
Intermediary metabolism	MT3949	bfrB	ferritin family protein	Rv3841	−3.178	−3.401	−1.809	−2.796	−6.944
	MT3969	ethA	monooxygenase, flavin-binding family	Rv3854c	−3.630	−2.818	−3.209	−3.219	−9.310
	MT3349	rubA	rubredoxin	Rv3251c	−2.793	−2.330	−2.688	−2.604	−6.078
	MT3348	rubB	rubredoxin	Rv3250c	−2.064	−1.773	−2.262	−2.033	−4.093
Cell wall associated	MT3169	lipR	acetyl−hydrolase	Rv3084	−2.807	−1.934	−1.114	−1.951	−3.867
	MT2912	efpA	efflux protein	Rv2846c	−1.691	−1.269	−1.243	−1.401	−2.641
	MT0870	lpqS	hypothetical protein	Rv0847	2.801	2.422	2.643	2.622	6.156
	MT1379	murI	glutamate racemase	Rv1338	3.229	3.500	3.325	3.351	10.205

Genes induced and repressed by functional category 90 minutes post-diamide treatment in the complemented strain. n = 3. MT # and Rv # denote the CDC1551 and the H37Rv gene IDs, respectively.

**Figure 5:**
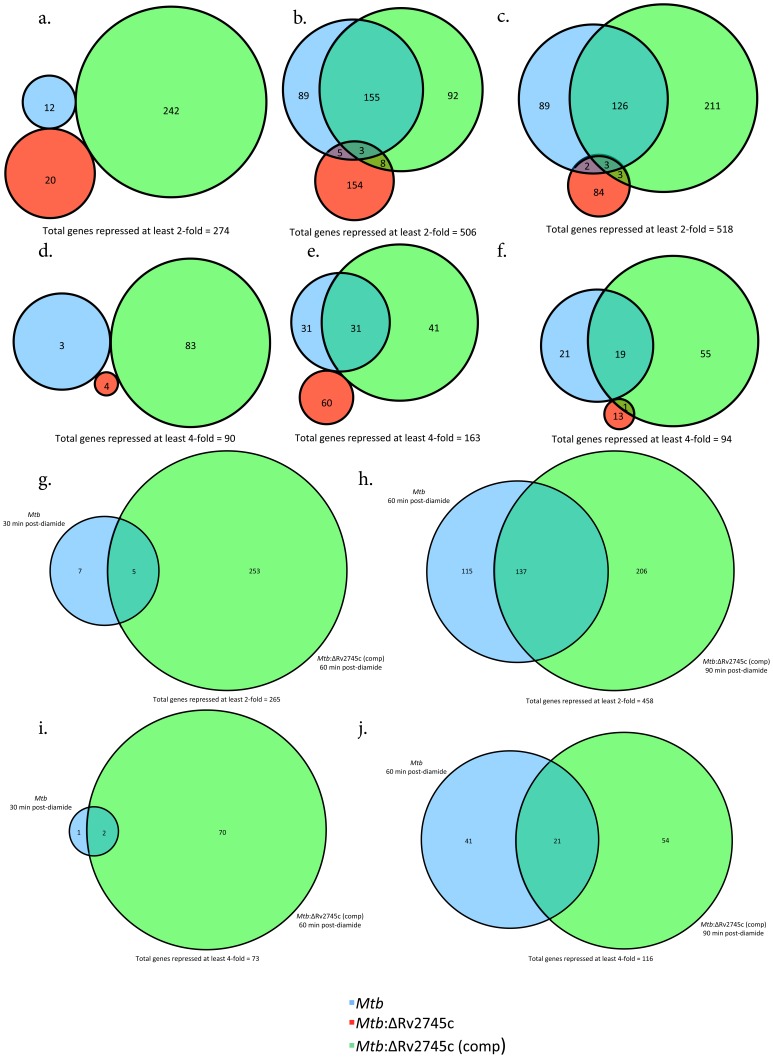
Venn Diagrams of Diamide Repressed Genes. Venn diagrams describe the extent of overlap between gene-expression upon diamide treatment in *Mtb* (blue circles), *Mtb*:ΔRv2745c (red circles) and *Mtb*:ΔRv2745c (comp) (green circles). Genes repressed at least two-fold at: a.) 30, b.) 60, and c.) 90 min post-diamide treatment are shown. Genes repressed at least four-fold at: d.) 30, e.) 60, and f.) 90 min post-diamide treatment. g–j.): Delayed response of *Mtb*:ΔRv2745c (comp). g.) Genes repressed at least two-fold comparing *Mtb* at 30 min to *Mtb*:ΔRv2745c (comp) at 60 min and h.) Genes repressed at least two-fold comparing *Mtb* at 60 min to *Mtb*:ΔRv2745c (comp) at 90 min. i.) Genes repressed at least four-fold comparing *Mtb* at 30 min to *Mtb*:ΔRv2745c (comp) at 60 min. j.) Genes repressed at least four-fold comparing *Mtb* at 60 min to *Mtb*:ΔRv2745c (comp) at 90 min. n = 3.

Profiles that exhibit repression, i.e. lower expression levels relative to baseline following redox stress were apparent at t = 60 min and t = 90 min rather than t = 30 min. Thus, of the 274 genes that are down regulated >2-fold at t = 30 min, there was no overlap in all three strains (Figure 5a). However, at t = 60 min, a significantly higher number of genes (506) showed repression >2-fold (Figure 5b). Of the 252 and 258 genes repressed >2-fold in *Mtb* and *Mtb*:ΔRv2745c (comp), respectively, 155 (62% and 60% respectively) were commonly repressed, whereas of the 170 genes repressed >2-fold in *Mtb*:ΔRv2745c only 3 were shared amongst all three strains (Figure 5b). This pattern was reflected at t = 90 min, wherein, of the 518 genes that are repressed >2-fold, 126 were shared between *Mtb* and the complemented strain (Figure 5c). A total of 220 and 343 genes were repressed >2-fold in wild-type and complemented strains, respectively, while only 92 genes were repressed >2-fold in the mutant (Figure 5c).

**Figure 6:**
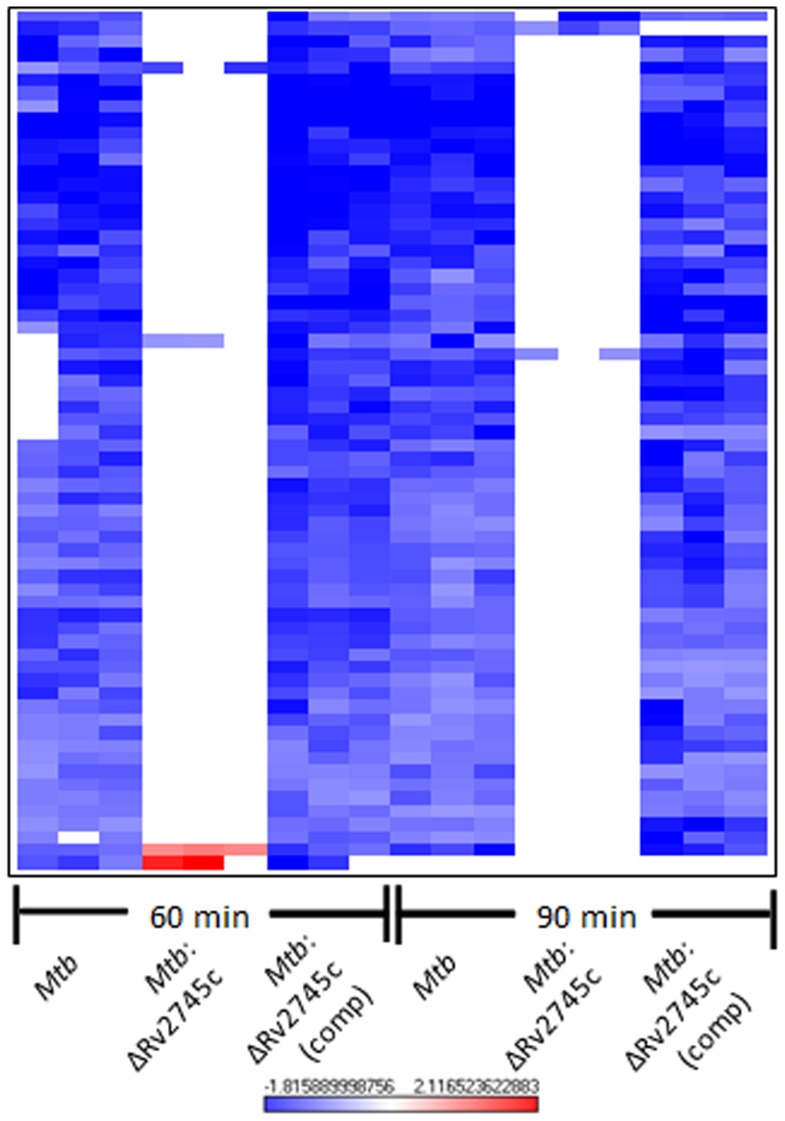
Diamide Repressed Genes. The heat map shows results of unsupervised hierarchial clustering focusing on genes with the greatest level of repression compared to the control channel. A majority of genes repressed in *Mtb* are also repressed in *Mtb*:ΔRv2745c (comp) at both 60 and 90 minutes post-diamide treatment, whereas there is no change in expression levels in *Mtb*:ΔRv2745c. n = 3. Red color indicates induction while blue color indicates repression, relative to the control channel. The intensity of each color corresponds to the magnitude.

This delayed response amongst genes that are repressed by >2-fold in the wild-type and complemented strains indicates that the repression of gene-expression is a secondary effect rather than a primary function mediated by the Rv2745c-encoded transcription factor (Figures 5 a–c, Figure 6, Tables 4, 6, 7, and 9). This implicates that the main role of clgR is to induce a subset of genes upon oxidative stress, and that some of these induced genes may eventually mediate or result in the repression of downstream genes. At both t = 60 min and t = 90 min, a majority of the genes that were repressed by at least two-fold were down regulated in both *Mtb* and *Mtb*:ΔRv2745c (comp) (Figure 5b & 4c).

To enhance the stringency of our analysis, we assessed the repression of genes in the three strains by >4-fold post-diamide treatment. A total of only 90 genes fulfilled this criterion at t = 30 min (Figure 5d). Of these, a majority (83) was repressed in the complemented strain and there was no overlap of shared repressed genes amongst the three strains (Figure 5d). The total number of repressed genes >4-fold increased to 163 at t = 60 min (Figure 5e). Of these, 31 were repressed in both wild-type and the complemented strain, whereas no genes in the mutant strain were also similarly repressed >4-fold in *Mtb* or the complemented strain at this time. (Figure 5e). At t = 90 min, the expression of a total of 94 genes was repressed >4-fold, of which 19 were commonly repressed in both wild-type and the complemented strain (Figure 5f). Of the 75 genes repressed >4-fold in the complemented strain, one was repressed in the mutant, as well (Figure 5f). The delayed response of the complemented strain that was seen in induced genes was not reflected in the repressed set of genes both in genes repressed >2-fold and >4-fold (Figures 5 g–j).

At 60 and 90 min post-diamide treatment the expression of ESAT-6 was repressed >2-fold in the wild-type and complemented strain, relative to its basal level of expression in the mutant strain (Tables 4, 5, 6, 7, 8, 9) as well as in all three strains at t = 30 min (Tables 1, 2, 3). This repression is most likely a secondary effect in response to the action of clgR induced genes that are upstream of ESAT-6 in the signaling cascade. Additionally, the levels of mprA were also repressed in both the wild-type and the complemented strain strains at both 60 and 90 min post-diamide treatment while remaining at basal levels in the isogenic mutant (Tables 4, 5, 6, 7, 8, 9).

When comparing repressed genes within *Mtb* and *Mtb*:ΔRv2745c (comp), a high degree of similarity emerges 60 and 90 min post-diamide treatment (Figure 6). However, this pattern of repression is ablated in the isogenic mutant, implicating that a secondary response occurs in which *clgR* activation of a subset of genes leads to repression of an additional subset of genes (Figure 6).

**Figure 7:**
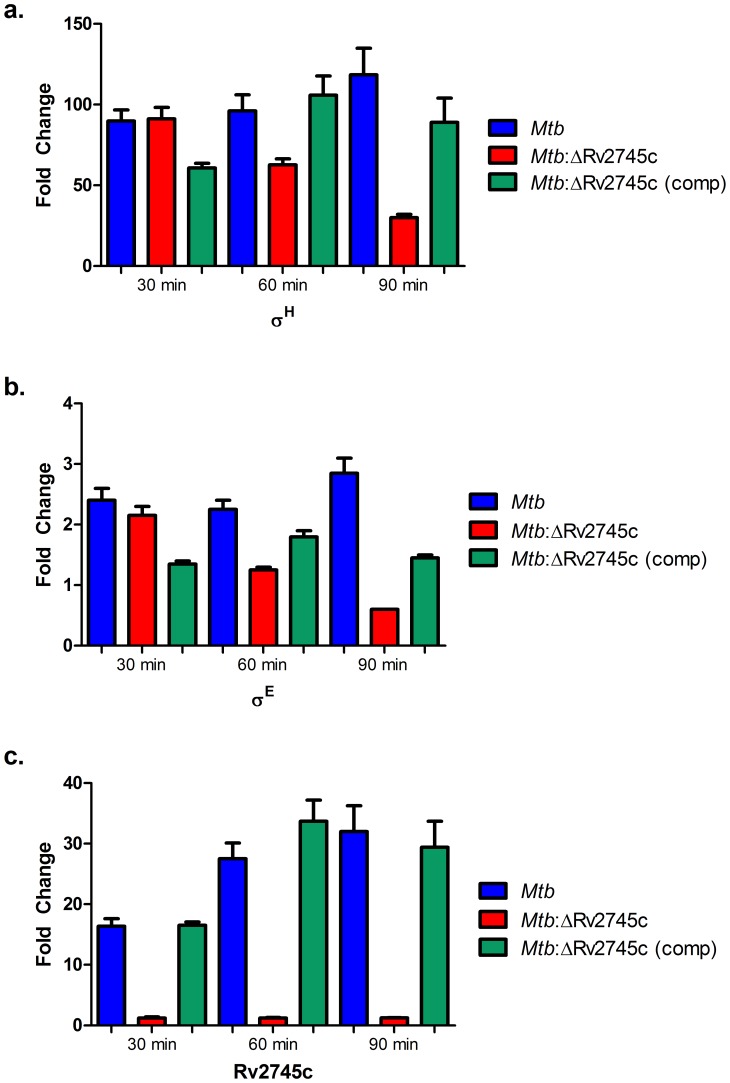
RT-PCR Confirmation. a.) The data shows that there is a small increase in σ^H^ levels of the isogenic mutant, relative to that of the wild type and complemented strain. However, σ^H^ levels are not sustained in the isogenic mutant, whereas they increase in the wild-type and complemented strain. b.) σ^E^ levels remain low in all three strains. c.) There is Rv2745c activation in the wild-type and the complemented strain, whereas there is no Rv2745c expression in the isogenic mutant, as expected.

Due to the extensive use of microarray technology in order to understand the transcriptomic networks that involve Rv2745c, we performed quantitative RT-PCR in order to confirm many of the salient results obtained using the microarray platform (Figure 7). RT-PCR confirmed that the expression of Rv2745c was not induced at any time point in the mutant, but was highly induced in both *Mtb* and the complemented stain at all time points, with levels of the transcript increasing in the complemented strain (Figure 7c). Furthermore, RT-PCR detected very low levels of σ^H^ induction in the isogenic mutant at the 30-minute time point (Figure 7a) similar to those observed in microarray experiments. Furthermore, the levels of σ^H^ transcript decreased throughout the time course in the isogenic mutant. RT-PCR revealed low levels of σ^E^ induction in each of the strains but the levels of this transcript significantly increased in *Mtb* and the complemented strains, relative to the mutant, over the course of time (Figure 7b).

## Discussion

The ability of *Mtb* to persist within host tissues for extended period of time indicates that this pathogen has developed unique mechanisms for its survival. Undoubtedly, some of these mechanisms involve coping with the various host and environmental stresses. *Mtb* is uniquely positioned to respond effectively to numerous stress conditions at it encodes numerous stress response transcription factors including sigma factors, two-component systems, eukaryotic like kinases etc. [26]. The proteins encoded by these diverse transcriptional and post-transcriptional regulators form specific stress-response regulons, which respond to a variety of environmental changes. The complexity of this system is enhanced by the interaction and interdependence of different stress regulons.

A key *Mtb* stress regulon is controlled by the expression of sigma factors, σ^H^, primarily in response to oxidative stress [16], [27]. In turn σ^H^ results in the induced expression of σ^E^, σ^B^ and a number of other transcription factors, thus directly and indirectly shaping the oxidative-stress dependent expression of over 500 *Mtb* genes [14]. Loss of σ^H^ ameliorates pathology in mice [27], reduces *Mtb* cfu levels in primate macrophages [28] and results in the complete lack of virulence in primates [29]. Thus, σ^H^ appears to be critical for *Mtb* to survive the host phagocyte oxidative burst. Expression of σ^E^ can be induced by σ^H^ as well as independent of it. During redox stress, modeled by diamide, the expression of both σ^H^ and σ^E^ is induced. Loss of σ^E^ also results in diminished virulence and pathology in the mouse model [30]. Rv2745c is predicted to encode a transcription factor with homology to Clp gene regulator (ClgR) in related gram-positive organisms [31]. The expression of Rv2745c is induced in response to numerous conditions that result in the up regulation of either σ^H^ or σ^E^ e.g. redox stress by diamide [14], cell envelope damage by thioridazine, vancomycin or SDS [19], [20], [32], low pH [6], enduring hypoxia and reaeration [18], etc. Interestingly however, this protein appears to perform different functions in many of these situations. Thus, Personne, *et al.* report that the expression of Clp protease genes clpP1, clpP2, clpC1 and clpX, known to be induced by ClgR upon subjecting *Mtb* to hypoxia, are not induced during redox stress [21]. Our group has similarly reported that while the expression of Rv2745c is induced >50-fold in *Mtb* following thioridazine treatment, the expression of the downstream clp genes was not induced [20]. Similarly, the expression of clp genes was also not induced by elevated levels of Rv2745c following vancomycin treatment of *Mtb* [32]. These results raise the possibility that the Rv2745c-encoded protein may indeed perform diverse functions in response to the different stress conditions, which result in the induction of its expression in *Mtb*. If this is true, then it may be possible that *Mtb* has adapted a stress response pathway present in related, saprophytic actinomyces to benefit its specialized pathogenic needs. It is possible that while retaining its function of inducing the expression of Clp proteases in response to hypoxia, this protein additionally also performs a moonlighting function in response to oxidative and envelope damaging stress. To specifically address this issue we have begun to modularly study the phenotype of the *Mtb*:ΔRv2745c mutant in response to these conditions. The current manuscript serves as the initial report in this regard. Here we demonstrate that the mutant is exquisitely sensitive to redox stress by diamide, which leads to rapid and strong induction of Rv2745c expression. We further show that the induction of Rv2745c does not result in the up regulation of Clp genes during redox stress. Instead, the expression of the σ^H^/σ^E^ network is dysregulated in the ΔRv2745c mutant.

Our results indicate that at least during redox stress due to diamide, the major function of Rv2745c induction in the absence of downstream clp gene induction may be to supplement the σ^H^/σ^E^ regulon. Hence, the *Mtb*:ΔRv2745c exhibits an *in-vitro* phenotypic susceptibility to diamide mediated oxidative stress, which is not observed for either *Mtb* or *Mtb*:ΔRv2745c (comp), implicating that *clgR* plays a role in mediated signaling cascades involved in response to redox stress. This altered phenotype is further supported by the differential expression patterns seen in the isogenic mutant when compared to both the wild type and complemented strain. Thus, the transcriptional phenotype of the *Mtb*:ΔRv2745c mutant in response to diamide stress *in-vitro* closely resembles those of the *Mtb*:Δ-σ^H^ and *Mtb*:Δ-σ^E^ mutants. Hence, the expression pathways, expression of σ^H^, σ^E^, σ^B^, the thioredoxin/thioredoxin reductase and the cysteine metabolic pathways was disrupted in the *Mtb*:ΔRv2745c mutant. It is conceivable, based on our data, that the Rv2745c-encoded protein is somehow involved in the reinforcement of the σ^H^/σ^E^ response to redox stress (Figure 6), perhaps by playing a regulatory role in the positive feedback loop that maintains σ^H^ or σ^E^ regulation [20], [22]. This role is independent of clp protease activation, indicating that Rv2745c may also directly activate σ^E^ and/or σ^H^ so that σ^H^ can mediate other downstream signaling events. This indicates that the positive feedback loop of Rv2745c with the downstream gene of the σ^H^ regulon, σ^E^, is more influential and plays a more prominent role in inducing the σ^H^ regulon than previously thought.

The acute dysregulation of the σ^H^/σ^E^ regulon, which involves genes upstream of *clgR*, as well as downstream genes in the sulfate assimilation pathway, occurred in *Mtb*:ΔRv2745c, implicates a strong alternative role for Rv2745c in signaling cascades responsible for responding to redox stress. This may be mediated through a direct binding of ClgR to either (or both) σ^H^ or σ^E^ promoter elements to reinforce the transcriptional signal or through an indirect effect of Rv2745c activation of clp proteases. This could be independent of the Clp protease complex. Accordingly, we did not detect an increase in clp protease (clpP1 as well as clpP2) transcriptional levels compared to t = 0 min, which is supported by the studies of Personne, *et al*., indicating that clpP activation is condition dependent [21]. However, we must acknowledge the possibility that Clp protease complex is indeed activated by ClgR during redox stress, albeit at lower levels such that the transcriptional induction of clp genes by either microarrays or RT-PCR is virtually undetectable. In this scenario, the enhanced activity of the protease complex could result in a higher turnover of RshA and RseA, the cognate anti-sigma factors for σ^H^ or σ^E^. In this regard, Sureka, *et al.* have shown that RseA is indeed a biological substrate for the *Mtb* clp protease system during stringent response [33]. Decreased availability of either anti-sigma factor could also potentially result in an over-exuberant expression of the σ^H^/σ^E^ network. Similarly, it is possible that the expression of a negative regulator of clp protease genes is induced during redox stress but not during hypoxia. Such an arrangement could result in the availability of ClgR during both conditions but the expression of clp protease system only during the latter situation. The expression profiles of a few known transcriptional repressors were found to be up regulated in *Mtb* vis-à-vis the mutant, during diamide stress (Table 10). However, unraveling the exact mechanism of this discrepancy will require more work.

**Table 10:** Negative Regulators Expressed in *Mtb* post-diamide treatment.

			Expression Fold-Change		
MT #	Description	Rv #	30 min	60 min	90 min
MT0073	transcriptional regulator, TetR family	Rv0067c	10.573	6.246	6.246
MT0206	transcriptional regulator, putative	Rv0196	2.364	5.363	5.363
MT0343	transcriptional regulator, TetR family	Rv0328	14.405	4.157	4.157
MT1079	transcriptional regulator, MarR family	Rv1049	20.578	8.545	8.545
MT3262	transcriptional regulator, TetR family	Rv3173c	3.198	2.802	2.802
MT3938	transcriptional regulator, TetR family	Rv3830c	16.089	3.924	8.074
MT3948	hypothetical protein	Rv3840	16.382	4.902	8.711

Probable negative transcriptional regulators induced in *Mtb* post-diamide at each time point. n = 3. MT # and Rv # denote the CDC1551 and the H37Rv gene IDs, respectively.

We also report higher expression levels of *katG* in *Mtb* at all time points upon redox stress, whereas the levels of *katG* remained unchanged in *Mtb*:ΔRv2745c (Tables 1, 2, 3, 4, 5, 6, 7, 8, 9). *katG* is a catalase peroxidase that is required for both countering peroxide products generated by NADPH oxidase of the phagocyte and activation of isoniazid, which is a key drug in TB treatment [34], [35]. However, in a study conducted by Mehra, *et al*., Rv2745c induction led to increased clp proteases and decreased levels of *katG* [10]. Hence, it is conceivable that *katG* may be a direct target of clpP proteases that are under the transcriptional control of *clgR*.

While the binding region of *Mtb clgR* has not yet been characterized, the consensus sequence for the clgR operator in *C. glutamicum*, *S. lividans*, and *Bif. breve* is well studied [11]. According to Estorninho, *et al*., there are several genes with a promoter-binding region specific to *clgR* when comparing consensus sequences, which support our result that Rv0384c (*clpB*), Rv3269 and Rv0251c were up regulated in the *Mtb* and not *Mtb*:ΔRv2745c at all time points (Tables 1, 2, 3, 4, 5, 6, 7, 8, 9) [11]. Rv0250c, which also has a *clgR* consensus sequence, was also up regulated in *Mtb* at two of the three time-points post-diamide treatment when compared to the untreated control (Tables 1 & 7) [11]. The Rv0251c – Rv0249c operon encodes for Acr2 (hsp), which is a chaperone that functions as an oxidoreductase and succinate dehydrogenase, whose regulation has been shown to be σ^E^ dependent upon SDS stress [11], [36]. Up regulation of *acr2* also occurs upon activation of σ^H^ and σ^E^ during oxidative stress, consequently *clgR* may also be responsible for the activation of *acr2*, as promoter binding sites for σ^H^ and σ^E^ are also found upstream of *acr2* [37].

A critical observation in our study was that while the complemented strain resulted in comparable expression of ClgR at the transcript as well as the protein level, and was able to rescue the mutant phenotype, at the molecular level, a significant delay existed in the complete complementation of the stress response. Thus, gene-expression at t = 60 min in *Mtb* mirrored that observed at t = 90 min for the complemented strain (Figure 2 h–j). These results reinforce the importance of using complemented strains in gene-functional evaluation experiments. Further, our results suggest that trans-complementation of bacterial genes could sometimes result in either a partial or, as observed in this instance, a delayed complementation of the phenotype. What effect this delay would have on the phenotype of the complemented strain *in-vivo*, remains to be seen. Such experiments are currently underway in our laboratory.

We also observed that genes whose expression was repressed in *Mtb* and the complemented strain (at either >2- or >4-fold limit) occurred only at later (t = 60 min & t = 90 min, but not t = 30 min) stages, indicating that secondary effect(s) are at play. It is likely that some of the genes induced directly in *Mtb* due to diamide stress cause the repression of other genes by either transcriptional or post-transcriptional mechanisms. Hence, down-regulation of *mprA* occurred in both wild-type and complemented strains while remaining at basal levels in the mutant at all time points, implicating an additional role for Rv2745c-encoded ClgR protein (Tables 4, 5, 6, 7, 8, 9). MprA can function as both an activator and a repressor of *acr2*, depending on condition [36]. However, the expression of *mprA* was repressed at both 60 and 90 min in the wild type and the complemented strain, indicating, that in the absence of MprA, Rv2745c may induce *acr2* [36]. MprA also induces the expression of σ^E^ independent of σ^H^ [38]. Thus, the induction of σ^E^ via σ^H^ due to redox stress apparently shuts down *mprA* transcription via feedback and it appears that Rv2745c plays a role in this process. The delay in repression is more than likely due a secondary response of repression, in which Rv2745c activated genes may be responsible for repression of downstream genes, which also explains why there is a general delay in down-regulation in the wild-type and complemented strains (Figure 6).

RT-PCR data revealed that there are low levels of σ^H^ induction in the isogenic mutant (Figure 7a). However, these levels continued to decrease throughout the course of diamide treatment, implicating that Rv2745c helps facilitate maintenance of the σ^H^ regulon upon application of redox stress. Additionally, the complemented strain and wild type *Mtb* had similar σ^H^ induction, which further supports that Rv2745c plays a role in the σ^H^ positive feedback loop. RT-PCR also confirms that Rv2745c was not induced in the isogenic mutant, but was induced to similar levels in both the wild-type and complemented strain (Figure 7c).

**Figure 8:**
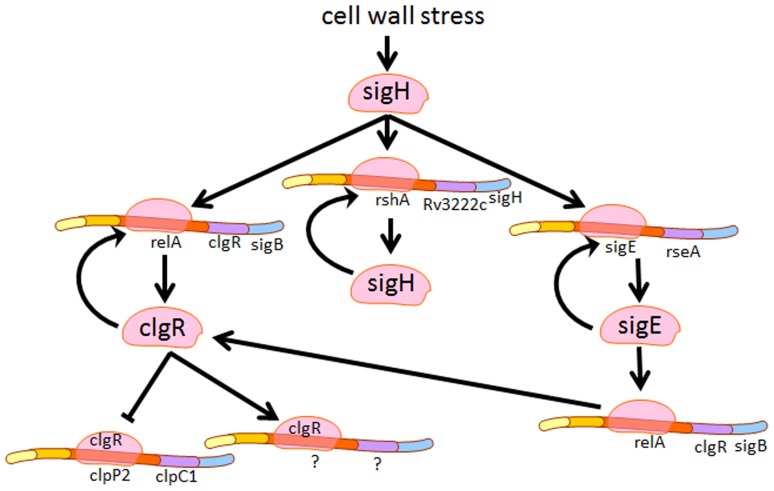
clgR activation schematic. Proposed overview of possible clgR activation upon cell wall stress. Induction of the σ^H^ regulon requires clgR activation. However, it is unclear if induction of the σ^H^ regulon is through direct or indirect effects of clgR activation.

Our results show that the Rv2745c-encoded protein facilitates key down-stream signaling in response to redox stress. Deletion of Rv2745c leads to disruption of key regulatory networks, such as the σ^H^ regulon, the cysteine biosynthetic pathway, and the thioredoxin pathways. Disruption of upstream genes, such as σ^H^ and σ^E^, implicate that Rv2745c may facilitate the positive feedback loop of this regulatory network either via direct transcriptional or an indirect post-transcriptional mechanism (Figure 8). Thus, further studies are required to clarify role of Rv2745c in the pathogenesis of *Mtb*. Further understanding of the function of Rv2745c in response to various environmental pressures may help lead to a better understanding as to how *Mtb* is able to survive and persist within the AMΦ.

## Supporting Information

Figure S1 **PCR Screening for** ***Mtb*** **:ΔRv2745c.** a. PCR using Rv2745c primers. Amplification of ∼339 base pairs in lane 4 shows the presence of Rv2745c in *Mtb* wild type, while its absence in lane 5 indicates deletion of Rv2745c from *Mtb*:ΔRv2745c. b. PCR amplification of hyg^r^. The absence of a band in lane 4 confirms the expected finding that hyg^r^ is not present within the *Mtb* genome. The presence of a band from hyg^r^ carrying plasmid as well as from genomic DNA derived from *Mtb*:ΔRv2745c is indicative of the replacement of Rv2745c by hyg^r^ (Lane 5). Lane 1–5(L–R): 0.1–12 kbp Ladder; Neg. Ctrl. (No DNA);ΔRv2745c::HygR phasmid; *Mtb* and *Mtb*:ΔRv2745c.
(TIF)

Figure S2 **Western Blot of Rv2745c levels.** Rv2745c protein levels after 60 minutes post-diamide treatment were detected via Western Blot. Whole cell lysates from several isolated colonies from the complementation were used. Rv2745c levels were restored to similar levels in the complemented strain relative to wild-type. From left to right, lane order: *Mtb*, *Mtb*:ΔRv2745c (comp, 2), *Mtb*:ΔRv2745c (comp, 4), *Mtb*:ΔRv2745c (comp, 10), *Mtb*:ΔRv2745c. Rv2745c levels were absent in the isogenic mutant 60-minutes post-diamide treatment.
(TIF)

Table S1 **Primers for PCR, Southern Blot, and RT-PCR.**
(PDF)
