# Supplementary figures and images for: The Mycobacterium tuberculosis Rv2745c Plays an Important Role in Responding to Redox Stress
Source: PLoS One. 2014 Apr 4;9(4):e93604. doi: 10.1371/journal.pone.0093604 (PMC3976341; doi:10.1371/journal.pone.0093604)

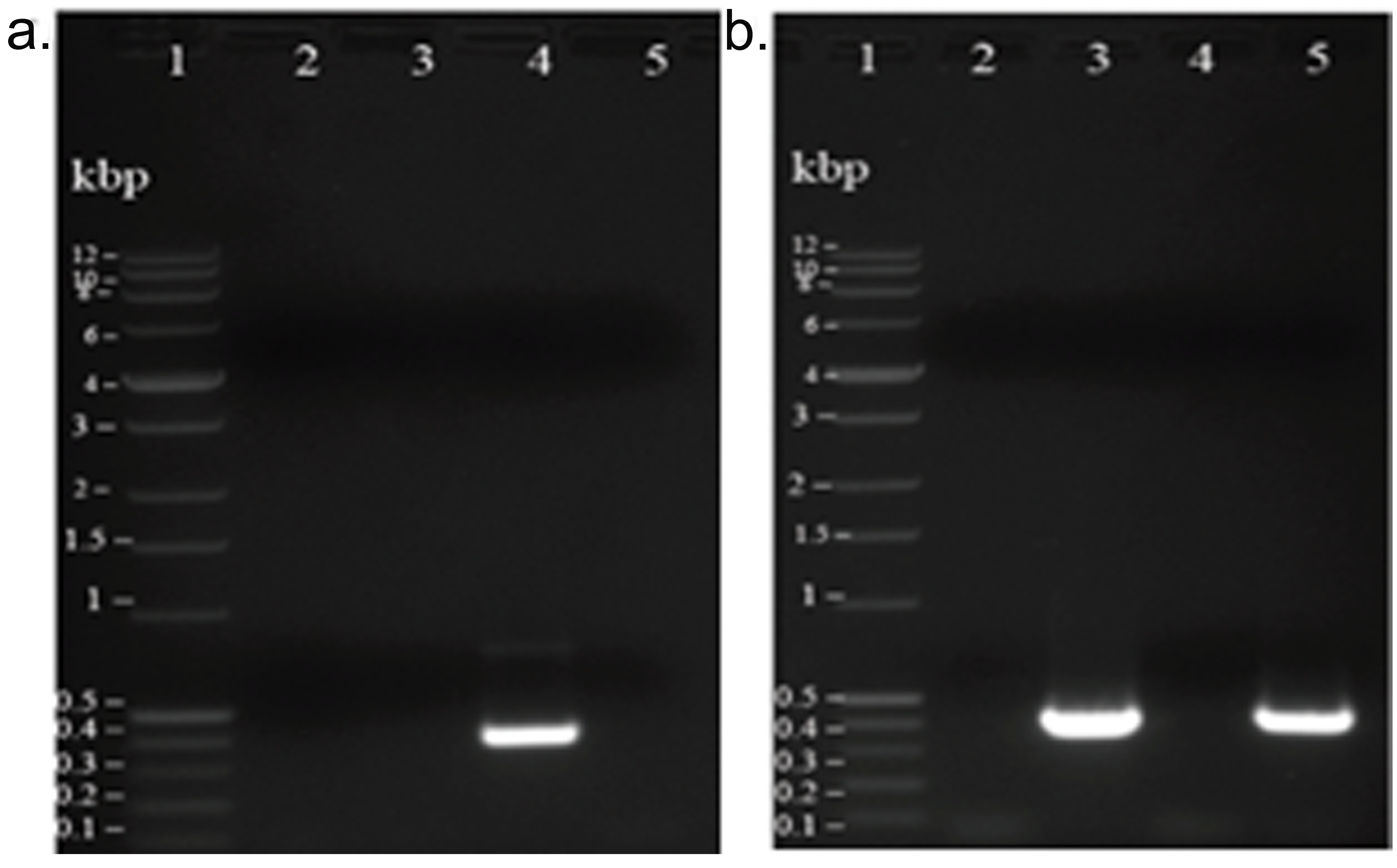

Supplement: Figure S1 — PCR Screening for Mtb :ΔRv2745c. a. PCR using Rv2745c primers. Amplification of ∼339 base pairs in lane 4 shows the presence of Rv2745c in Mtb wild type, while its absence in lane 5 indicates deletion of Rv2745c from Mtb:ΔRv2745c. b. PCR amplification of hygr. The absence of a band in lane 4 confirms the expected finding that hygr is not present within the Mtb genome. The presence of a band from hygr carrying plasmid as well as from genomic DNA derived from Mtb:ΔRv2745c is indicative of the replacement of Rv2745c by hygr (Lane 5). Lane 1–5(L–R): 0.1–12 kbp Ladder; Neg. Ctrl. (No DNA);ΔRv2745c::HygR phasmid; Mtb and Mtb:ΔRv2745c. (TIF) [file pone.0093604.s001.tif]

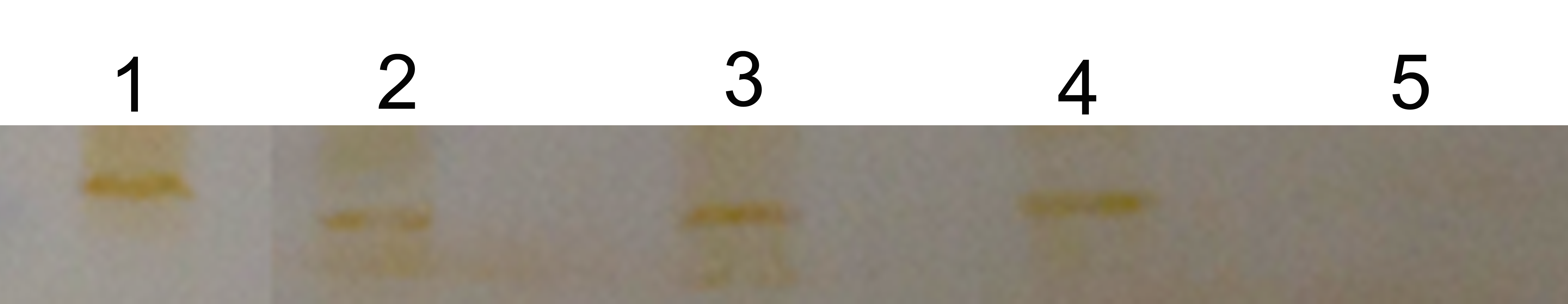

Supplement: Figure S2 — Western Blot of Rv2745c levels. Rv2745c protein levels after 60 minutes post-diamide treatment were detected via Western Blot. Whole cell lysates from several isolated colonies from the complementation were used. Rv2745c levels were restored to similar levels in the complemented strain relative to wild-type. From left to right, lane order: Mtb, Mtb:ΔRv2745c (comp, 2), Mtb:ΔRv2745c (comp, 4), Mtb:ΔRv2745c (comp, 10), Mtb:ΔRv2745c. Rv2745c levels were absent in the isogenic mutant 60-minutes post-diamide treatment. (TIF) [file pone.0093604.s002.tif]
