# Supplementary material for: The Mycobacterium tuberculosis Rv2745c Plays an Important Role in Responding to Redox Stress
Source: PLoS One. 2014 Apr 4;9(4):e93604. doi: 10.1371/journal.pone.0093604 (PMC3976341; doi:10.1371/journal.pone.0093604)
Supplement: Table S1 — Primers for PCR, Southern Blot, and RT-PCR. (PDF) [file pone.0093604.s003.pdf]

Supplemental Table 1. Primer Sequences

Primer	Sequence (5' to 3')
Mtb Rv2745c Fwd	5' - GCATGCGACGATTGCGAGGTGGACAA –3′
Mtb Rv2745c Rev	5' - GGCCTTAATTAATTAGGCCAGCGCCA –3′
Hyg^r Fwd	5' - CCATTCCGAGGTCTTCCCGGAACTGC –3′
Hyg^r Rev	5' - CCTCGAACACCTCGAAGTCGTGCAGC –3′
Mtb sigA Fwd RTPCR	5' - CCTGGGAAACTGGGTCTAATAC - 3'
Mtb sigA Rev RTPCR	5' - CTCATCCCACACCGCTAAAG - 3'
Mtb Rv2745c Fwd RTPCR	5' - CCTCGGGTATCTGTCGGAGAT -3'
Mtb Rv2745c Rev RTPCR	5' - AGCTGCAGAGCCGTACAAATC - 3'
Mtb Rv2744c Fwd RTPCR	5'- TCTCCAATTGACGCTGGTTACCGA -3'
Mtb Rv2744c Rev RTPCR	5'- CAAGGTGCAGATTCAACAGGCCAT -3'
Mtb Rv2743c Fwd RTPCR	5'- GCGGTGACCATTTCGTTGTACTGA -3'
Mtb Rv2743c Rev RTPCR	5'- TCGTATCTGGTGCCGACCATCAA -3'
Mtb sigH RTPCR Fwd	5'- GCCTATGCGGGATTTCGTT -3'
Mtb sigH RTPCR Rev	5'- GGTGTTGGTCAGTATCCGGTAGA -3'
Mtb sigE RTPCR Fwd	5'- ATGGAACTCCTCGGCGGA -3'
Mtb sigE RTPCR Rev	5'- CAATTGGTCAGACGGCTCCA -3'
Mtb moeB1 Fwd RTPCR	5' -CGGCGGCCAGATACAATAA - 3'
Mtb moeB1 Rev RTPCR	5' - AGCCGCCATCTCATCATTC - 3'
Mtb MT3140 Fwd RTPCR	5' - TGTGTCGATGTCTTCGTTGTAG - 3'
Mtb MT3140 Rev RTPCR	5' - GTCGTTCAACCGCCAGAT - 3'
Mtb trxB1 Fwd RTPCR	5' - GACATGGTGCTCGTCGATTAT - 3'
Mtb trxB1 Rev RTPCR	5' - CAAGCTCTCGTTCGGCTT - 3'
Mtb mec+ Fwd RTPCR	5' -GGCATATCCCGATGACCAAT - 3'
Mtb mec+ Rev RTPCR	5' - GCAGTGTGCGAGTGATAGAT - 3'
Mtb clpP1 Fwd RTPCR	5' - AAACCGTATTCCAGGGCTTC - 3'
Mtb clpP1 Rev RTPCR	5' - CGCCGTGATCAAGAAAGAAATG - 3'
Mtb clpC1 Fwd RTPCR	5' -TGCCCAGATTGGACGTAAAG - 3'
Mtb clpC1 Rev RTPCR	5' - CGCATCAGGAGATCTACAACAG - 3'
Mtb trxB2 Fwd RTPCR	5' - CTTCGAGGGCACGTCTTT - 3'
Mtb trxB2 Rev RTPCR	5' - CGCATCTCATCCATCAACTCT - 3'

Primer	Sequence (5' to 3')
Mtb cysN Fwd RTPCR	5' - CATATCAGCGGCCCAAGAA - 3'
Mtb cysN Rev RTPCR	5' - ATGACTCCAACAGGTCGATAAC - 3'
Mtb cysM Fwd RTPCR	5' - ATACTGACCGCGCGATATTC - 3'
Mtb cysM Rev RTPCR	5' - TTGAGATGCCCGCAAAGA - 3'
Mtb moaC2 Fwd RTPCR	5' - CCGTCTACCTTGAGGATGATTT - 3'
Mtb moaC2 Rev RTPCR	5' - GGTGGCATAGCGGAATAAGA - 3'
Mtb dnaK Fwd RTPCR	5' - CCTGAAGACACGCTGAACAA - 3'
Mtb dnaK Rev RTPCR	5' - CCGACTTGATGGCCGAAATA - 3'
Mtb clpB Fwd RTPCR	5' - TCGGCCAACTTCTCTTTCTG - 3'
Mtb clpB Rev RTPCR	5' - ATGGCGCTGTCCAAAGAA - 3'
Mtb kdpE Fwd RTPCR	5' - CCAGATCGACGGTGAATGAA - 3'
Mtb kdpE Rev RTPCR	5' - TGGAATGGACGAGTTTCTGG - 3'
Mtb hspR Fwd RTPCR	5' - GCGTACCTACGATCGTCTTG - 3'
Mtb hspR Rev RTPCR	5' - CGACGTCATGCAGGGAATAG - 3'
Mtb Rv3220c Fwd RTPCR	5' - CCGTCTGCAGGTTGTTCTTA - 3'
Mtb Rv3220c Rev RTPCR	5' - ATATTGATCCGCGACGTGAC - 3'
Mtb rshA Fwd RTPCR	5' - CCCTGCTTGACGGTGAAT - 3'
Mtb rshA Rev RTPCR	5' - GTTCCTCGAGCCCGTAATG - 3'
Mtb rseA Fwd RTPCR	5' - AGCACGTTGCTCGGATTAC - 3'
Mtb rseA Rev RTPCR	5' - GAACCTCCAGACGAACCTTTAG - 3'
